# Driven Radical Motion Enhances Cryptochrome Magnetoreception: Toward Live Quantum Sensing

**DOI:** 10.1021/acs.jpclett.2c02840

**Published:** 2022-11-04

**Authors:** Luke D. Smith, Farhan T. Chowdhury, Iona Peasgood, Nahnsu Dawkins, Daniel R. Kattnig

**Affiliations:** Living Systems Institute and Department of Physics University of Exeter, Stocker Road, Exeter EX4 4QD, United Kingdom

## Abstract

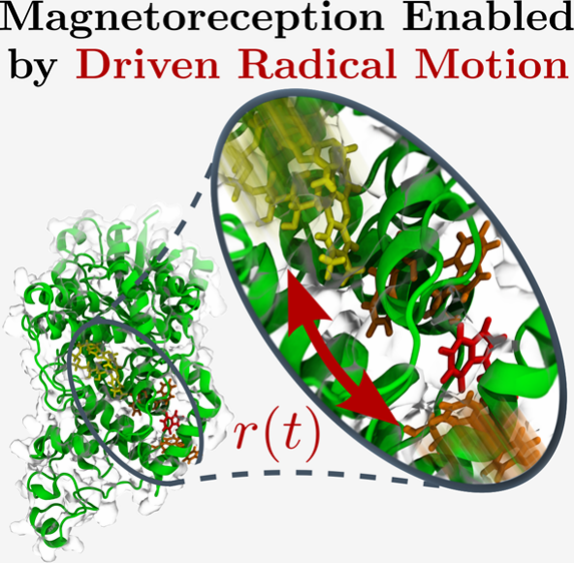

The mechanism underlying magnetoreception has long eluded explanation. A popular hypothesis attributes this sense to the quantum coherent spin dynamics and spin-selective recombination reactions of radical pairs in the protein cryptochrome. However, concerns about the validity of the hypothesis have been raised because unavoidable inter-radical interactions, such as the strong electron–electron dipolar coupling, appear to suppress its sensitivity. We demonstrate that sensitivity can be restored by driving the spin system through a modulation of the inter-radical distance. It is shown that this dynamical process markedly enhances geomagnetic field sensitivity in strongly coupled radical pairs via Landau–Zener–Stückelberg–Majorana transitions between singlet and triplet states. These findings suggest that a “live” harmonically driven magnetoreceptor can be more sensitive than its “dead” static counterpart.

The geomagnetic field provides a frame of reference that living systems use toward essential functions.^1,2^ Migratory birds exemplify this in their reliance on an internal compass that aids their navigation to breeding and wintering sites.^3^ Although what underlies this fine-tuned compass sense remains unsettled,^4^ growing evidence suggests its reliance on magnetosensitivity acquired through the quantum spin dynamics of a radical pair recombination reaction mediated by the blue-light-sensitive flavoprotein *cryptochrome*.^5,6^ However, many open problems remain, including the identity of the relevant in vivo radical pair. Involving the quantum dynamics of two spatially separated unpaired electrons,^7−10^ the widely studied radical pair mechanism (RPM) forms the cornerstone of the quantum compass hypothesis. Their combined spin angular momentum can be described in terms of singlet/triplet states that have distinct recombination reactions giving rise to different chemical products. Consequently, magnetosensitivity is elicited as a result of coherent singlet–triplet interconversion occurring predominantly due to hyperfine couplings of electron spins with surrounding magnetic nuclei and their interaction with an applied magnetic field. In cryptochrome, a commonly adopted model supported by in vitro studies assumes that a photoinduced electron transfer forms a radical pair between a flavin anion radical (FAD^•–^) and a tryptophan radical cation (TrpH^•+^).^11−13^ Alternative reaction mechanisms, such as dark-state oxidation schemes, have also been proposed and investigated.^14,15^

Theoretical studies have provided deeper insight, such as the inter-relation of coherence and magnetosensitivity, but are limited in more realistic settings,^16^ where the presence of many hyperfine couplings and environmental noise can constrain coherence lifetimes to a few microseconds.^11,17−20^ Furthermore, inter-radical couplings, such as electron–electron dipolar (EED) and exchange interactions, can suppress magnetosensitivity,^21−24^ but were neglected in a majority of theoretical studies. While the exchange interaction was shown to be negligible for selected cryptochromes via time-resolved EPR spectroscopy,^25^ due to the close vicinity of the recombining radical centers (approximately 1.5 nm) EED coupling is unavoidable.^24,26^ To resolve this, a mutual compensation of exchange and EED interactions was suggested,^26^ but it was found to be ineffective for radical pairs involving the flavin radical.^24^ The quantum Zeno effect was also proposed as a resolution,^27^ but this requires fast triplet recombination, which is arguably not physically realizable in cryptochrome. While alternate three-radical models,^28,29^ by suitable placement of an inert radical bystander^24,30^ or by a scavenger radical,^31^ can show enhanced magnetosensitivity despite the presence of EED coupling, they currently lack directly supporting experimental evidence.

It has been established that stochastic fluctuations of inter-radical distances due to (equilibrated) molecular dynamics can demonstrate improved magnetic sensitivity through spin relaxation in the Markovian limit.^32^ However, the question remains if enhancements could arise from driving, i.e., structured molecular dynamics that imprint a time dependence on the inter-radical separation. Here, we use “driving” to indicate the presence of a strong deterministic component in the distance modulation. Such motion could emerge intrinsically (e.g., as a result of relaxation oscillations in bistable systems or during the course of relaxation of a nonequilibrium system) or be actively maintained or even realized via potentially artificial means. A common feature is the non-Markovian character of the resulting spin dynamics resulting from the motion. In the context of entanglement, Cai, Popescu, and Briegel have previously associated similar driving with a class of “live” quantum phenomena that are persistent, are dynamically controllable, and exist only while metabolic processes take place, i.e., while the system is maintained far from thermal equilibrium in open driven systems.^33−35^ Here we retain this definition for “live” magnetoreception. To investigate the possibility of enhancements arising from driven radical motion, under this definition, we extend on the established RPM to incorporate inter-radical distance modulation, approximated as harmonic motion that modulates the exchange/EED interactions and the recombination rate, to find that the magnetic field sensitivity can be vastly amplified.

## Driven Model of Magnetoreception

In our model, radical pairs undergo coherent evolution subject to time-dependent harmonic driving of the inter-radical separation, with the Hamiltonian  comprising Zeeman, hyperfine, and time-dependent exchange and EED interactions. Following the idea of a reductionist approach, our model has been idealized by assuming periodic harmonic driving. While real proteins may not respond, e.g., to charge separation, in this exact manner, our motivation is to identify fundamental enhancing properties of driven radical motion that could potentially be harnessed in technology or mediated by dynamics in (artificial and natural) spin systems by actively maintaining a structured reaction coordinate. The simplicity of the model also enables efficient computer simulations via Floquet theory, which allows a systematic evaluation. More realistic models involving damped motion and underdamped Brownian motion are discussed below.

**Figure 1:**
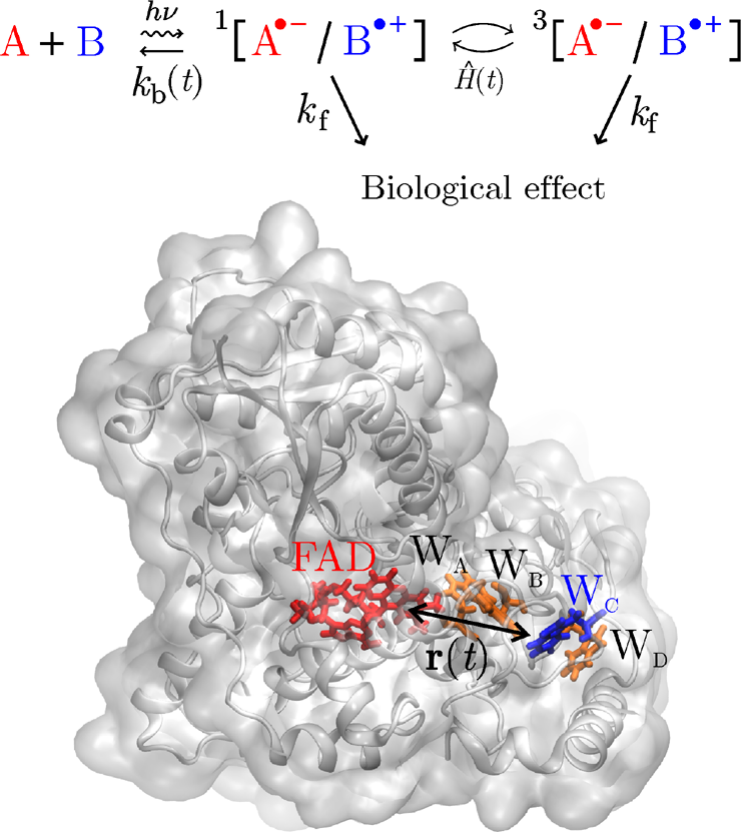
(Top) Reaction scheme imprinting magnetic field sensitivity on the recombination yield as a consequence of coherent singlet–triplet interconversion in a radical pair comprising A^•–^ and B^•+^. (Bottom) Structure model of a cryptochrome^36^ highlighting the FAD cofactor and the conserved electron transfer pathway formed from four tryptophan residues (W_A_, W_B_, W_C_, and W_D_). The FAD^•–^/W_C_^+•^ pair is one of the radical pairs proposed to underpin light-dependent magnetoreception. Driving of the inter-radical distance *r*(*t*) can enhance the magnetosensitivity, as demonstrated in this study.

The radical pair reaction of A^•–^ and B^•+^ involves singlet ^1^[A^•–^/B^•+^] and triplet ^3^[A^•–^/B^•+^] interconversion, recombination with rate *k*_b_(*t*) and forward reaction with rate *k*_f_ (Figure 1).

The initial singlet state of the reaction is given by a spin density operator , where  is the singlet projection operator and *Z* = *Z*_A_*Z*_B_ denotes the dimension of the nuclear subspace associated with the two radicals. The time evolution of  is described by the master equation
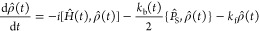
1where [ ] represents the commutator and {} the anticommutator. The solution to eq 1 given by , where the time evolution operator is
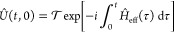
2with an effective Hamiltonian given by
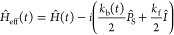
3The time-dependent recombination yield due to the singlet channel is found using
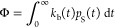
4where  and the singlet recombination yield is evaluated by propagating wave functions using , summed over all *i* initial singlet states of various nuclear spin configuration. As the effective Hamiltonian is periodic in time, i.e.,  with  denoting its period, we utilize Floquet theory to speed up the computations for large driving frequencies *k*_f_, *k*_b_ ≪ ν_d_ (see Supporting Information (SI) for more details).

To exemplify key features of a driven radical pair system, we first focus on a simple model comprising a single hyperfine-coupled nitrogen atom  in one radical and no hyperfine interactions in the other. Inter-radical interactions are considered in the form of a scalar coupling *J*(*r*) formally corresponding to the exchange interaction, but qualitatively also encompassing unavoidable EED coupling. For inter-radical distance modulated as
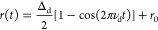
5we choose a singlet recombination rate of the form^37^
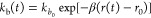
6with *r*_0_ = 17.8 Å specifying the inter-radical distance of the static radical pair,  = 2 μs^–1^, *k*_f_ = 1 μs^–1^, and β = 1.4 Å^–1^.^38^ Exchange interaction is taken in the same functional form
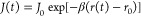
7The single nonzero hyperfine interaction was assumed to be axial, with principal components given by *A*_*xx*_ = *A*_*yy*_ = *A*_⊥_ = −2.6 MHz and *A*_*zz*_ = *A*_*∥*_ = 49.2 MHz, representative of the dominating nitrogen atom (N5) in flavin radicals. To assess directional magnetic field effect (MFE), we compute the relative anisotropy
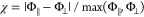
8where Φ_∥_ and Φ_⊥_ are the singlet recombination yields, calculated via eq 4, for the static magnetic field pointing in parallel and perpendicular directions, respectively.

**Figure 2:**
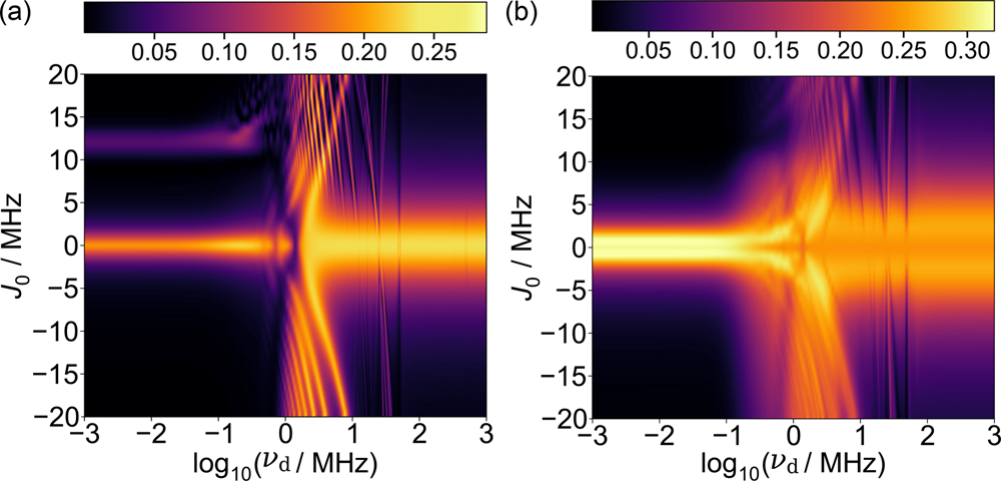
Color maps of the driven radical pair model with EED interaction neglected for a variation of exchange interaction strength *J*_0_ against driving frequency ν_d_. (a) Relative anisotropy χ. (b) Relative entropy of coherence *C*_r_ evaluated in the singlet–triplet basis. Values of |*J*_0_| ≳ 1 MHz create a suppressive effect in the static case, which is removed by including driving in the approximate range of 0 < ν_d_ < 10 MHz.

Figure 2a shows relative anisotropy for a variation of exchange interaction strength *J*_0_ against driving frequency ν_d_ and Δ_d_ = 3 Å. For the static case, inter-radical coupling is seen to suppress magnetic field sensitivity for values of |*J*_0_| ≳ 1 MHz. However, as driving frequency is increased, this suppression is lifted. In particular, a driving frequency in the approximate range of 1–10 MHz provides recovery of a MFE for −20 ≤ *J*_0_ ≤ 20 MHz (but extends to even larger values of |*J*_0_|; see SI). In Figure 2b, we take a time-integrated average of the commonly used relative entropy of coherence,^39^ defined as
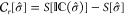
9over parallel and perpendicular directions of the magnetic field with the normalized density operator  chosen with respect to the electronic singlet–triplet basis  of the *d*-dimensional Hilbert space. Here, the dephasing operation is given by  and the von Neumann entropy is denoted by . The broad agreement between panels a and b of Figure 2 shows that a reinstatement of the MFE is accompanied by a stimulation of coherence, but this relation depends on synchronization with singlet–triplet oscillations of the driven system. We also considered alternative coherence and entanglement measures, with data available in the SI, to provide further explanation of specific features.

To elucidate the physical basis of the observed enhancements, we consider the case of the one-nitrogen radical pair with *A*_⊥_ = 0. With this simplification, the effective Hamiltonian is reducible with blocks labeled by the magnetic quantum number of the nuclear spin *m*_*I*_ ∈ {1, 0, – 1}. Using basis states |*T*_+_⟩, |*T*_0_⟩, |*T*_–_⟩, and |*S*⟩ associated with the triplet and singlet states, respectively, the system Hamiltonian for the magnetic field pointing along the perpendicular axis takes the form
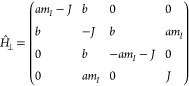
10where *a* = *A*_∥_/2 and ; a similar expression applies for the parallel orientation (see SI). The scenario encoded in these representation matrices resembles a Landau–Zener–Stückelberg–Majorana (LZSM) transition,^40−42^ where the relevant states of the avoided crossing are |*S*⟩ and |*T*_0_⟩ that have a constant coupling via the hyperfine interaction for *m*_*I*_ ≠ 0.

If inter-radical coupling is static and large, |*J*| ≫ *A*_∥_, then the energy separation of |*S*⟩ and |*T*_0_⟩ traps the system in the singlet state. Driving this system through a modulation of the inter-radical distance introduces a time-dependent *J*(*t*), decreasing it from *J*(*t*) = *J*_0_ at *t* = *n*/ν_d_ to *J*(*t*) = *J*_0_ exp(− βΔ_d_) at *t* = (2*n* + 1)/2ν_d_ for , thereby driving the system through avoided crossings for *a* ∼ *J*(*t*). In turn this induces LZSM transitions from |*S*⟩ to |*T*_0_⟩, which can be described as adiabatic evolutions interrupted by diabatic transitions as the system transitions through the anticrossings.^41^ The adiabatic phases accumulated between transitions can constructively or destructively interfere in the subsequent diabatic transition event, leading to the observed resonance effects as *J*_0_ and ν_d_ are varied. Consequently, the trapped singlet population is released which, in the perpendicular orientation, allows evolution to occur between |*T*_±_⟩ states mediated by the magnetic field and without suppression from exchange interaction. This is due to the common energy shift of the triplet states under exchange coupling. Similar LZSM transitions between |*S*⟩ and |*T*_0_⟩ occur for ; however, at this field orientation there is no further evolution to the |*T*_±_⟩ states. In the case of inter-radical coupling that is static and moderate, |*J*| ∼ *A*_∥_, the system dynamics lacks a significant MFE as it is characterized by fast |*S*⟩ – |*T*_0_⟩ interconversion at both orientations. Nevertheless, harmonic driving introduces a periodic reduction of *J* which modulates the interconversion amplitude, brings the system closer to the case unperturbed by *J*, and thereby enables population redistribution to |*T*_±_⟩ states for the perpendicular field orientation that reinstates magnetosensitivity.

Overall, these processes restore the MFE at a range of large to moderate *J*_0_ for particular driving frequencies. Although here it is further complicated by nuclear hyperfine interactions, in the SI we confirm that general enhancement features persist for a further simplified two-level system which, under a small amplitude expansion of the driving, is akin to the model considered by Shevchenko et al.^41^ (see Figure S8). The basis of this principle extends to systems with more hyperfine couplings and *A*_⊥_ ≠ 0. Further analysis on simple models may be found in the SI, where we show the case where the time-dependent recombination rate constant is substituted by its temporal average.

The driving in natural systems may be constrained, such as in charge separation initiated structural rearrangements in a protein leading to radical pair generation.^13,43^ To address this, we analyzed a model using damped oscillations and found that the MFE may be even further enhanced (Figure S9), suggesting the importance of driving in the early time spin dynamics for freeing the trapped singlet population through avoided crossings. Subsequently a decay in oscillation amplitude aids in enabling efficient recombination at suitable time periods. Additionally, we have used an optimal quantum control approach to maximize the directional sensitivity of the system. For exchange coupling *J*_0_ = 10 MHz, optimizing the absolute anisotropy gives enhancements up to χ = 0.73, exceeding effects realizable through harmonic driving by a factor of 3 (see Figure S10).

Thus far, a restoration of magnetic field sensitivity, due to driving, has been observed for a model system with axial symmetry subject only to the scalar exchange interaction. It could be argued that the observed enhancement is only due to the intermittent reduction of *J*_0_, which has a strong dependence on the distance and could be nullified by relatively small amplitude oscillations. In contrast, the EED interaction decays slowly with respect to *r* (∝1/*r*^3^) and could be more detrimental to magnetosensitivity. Therefore, we analyze the system again with EED interaction included, using parameters reflecting the relative orientation of the FAD and TrpH radicals in cryptochrome. As this system lacks axial symmetry, the MFE was assessed by the established measure , where Φ_min_ and Φ_max_ are the minimum and maximum yields and  denotes the average over orientations of the applied magnetic field relative to the spin system (assessed for 2562 orientations).

**Figure 3:**
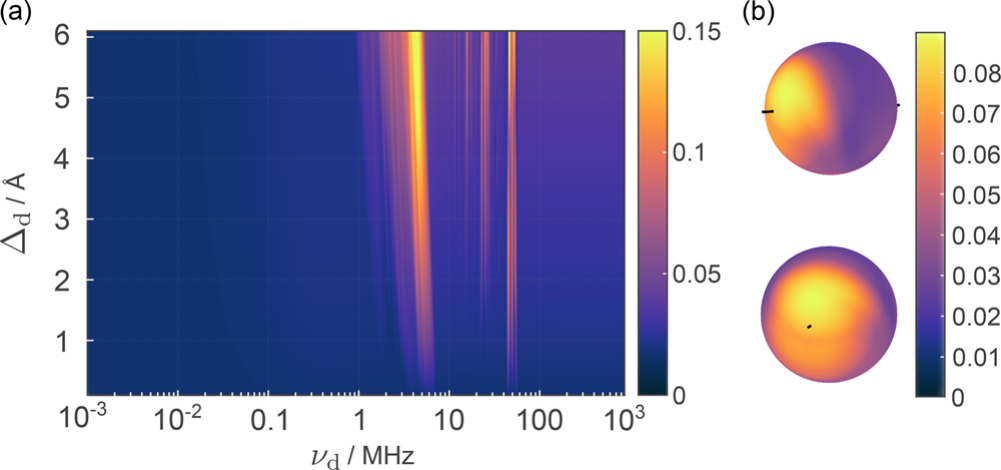
Driven radical pair model with EED interactions included, where *D*(*r*_0_) = −13.7 MHz, and *J*_0_ = 0. (a) Color map of the relative anisotropy. χ is shown for a variation of oscillation amplitude Δ_d_ against driving frequency ν_d_. The displacement is assumed to happen along the inter-radical axis. Relative anisotropy is suppressed in the static case, but can be restored for driving frequencies in the approximate range of 1 ≤ ν_d_ ≤ 100 MHz. (b) Orientation dependence is displayed for an oscillation amplitude of Δ_d_ = 2 Å, and ν_d_ = 4.4 MHz, demonstrating effectiveness with oscillations broadly along the inter-radical axis.

For *J*_0_ = 0, the case without additional exchange coupling, Figure 3a demonstrates that magnetosensitivity can be restored for a range of oscillation amplitudes Δ_d_ at an appropriate driving frequency, although the effect is more striking for larger amplitudes. We also observe a small enhancement in magnetosensitivity for oscillations that reduce the inter-radical distance (Figure S12). Consequently, the MFE persists as long as there is some driving that creates a time-dependent relative decrease in the EED in tandem with the change in recombination rate. In Figure 3b dependence of the effect on oscillation direction relative to the inter-radical axis is analyzed with Δ_d_ = 2 Å. We observe that an exact alignment is not required and oscillations that broadly increase the distance are effective. Oscillation amplitudes of 4 Å and 6 Å have been considered in the SI (Figure S11), which support these findings, showing that for increased oscillation amplitude the optimal oscillation direction is one that increases the inter-radical distance and is close to the inter-radical axis.

**Figure 4:**
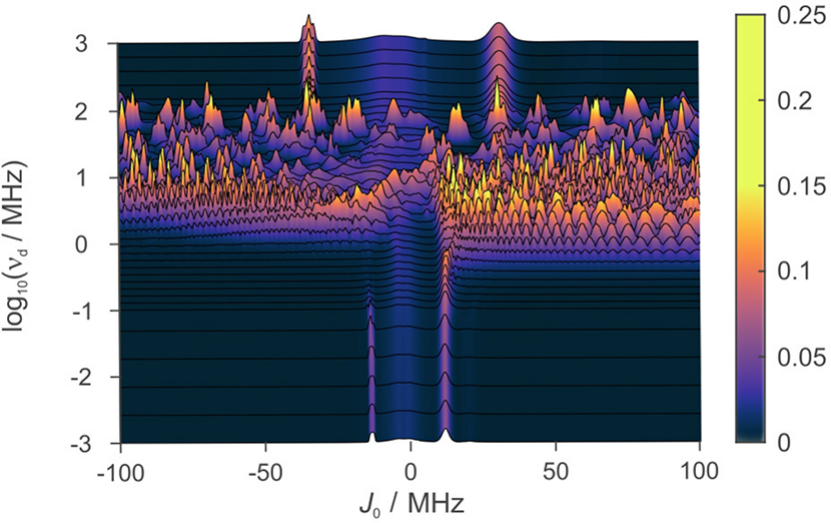
Color map for anisotropy of a driven model with both exchange and EED interactions, where *D*(*r*_0_) = −13.7 MHz, shown for variation of driving frequency ν_d_ against exchange interaction *J*_0_. The displacement is assumed to happen along the inter-radical axis. Restorations of anisotropy are observed for driving frequencies in the approximate range of 1 ≤ ν_d_ ≤ 100 MHz, even for large exchange interaction strengths in the range −100 ≤ *J*_0_ ≤ 100 MHz.

To analyze whether a MFE is possible in driven systems that are subject to both exchange and EED interactions, we have used the above model with exchange and EED included. In Figure 4 we show the results of this model for a variation of *J*_0_ in the range of −100 ≤ *J*_0_ ≤ 100 MHz and a choice of a relatively small oscillation amplitude of only Δ_d_ = 2 Å. In the static case, we observe two peaks of magnetosensitivity as a function of *J*_0_. As the system is driven in the range of 1–100 MHz, complex patterns emerge with markedly enhanced MFEs and a remarkable resilience to large *J*_0_. Within this range, there is a repetitive, though not strictly periodic, dependence on *J*_0_. As the driving frequencies are increased beyond 100 MHz, the response eventually resembles that of the static case, but with peaks occurring at larger *J*_0_ such that the time-averaged *J*(*t*) corresponds to the quasi-static *J*_0_.

## Magnetic Field Effect in Larger Driven Systems

**Figure 5:**
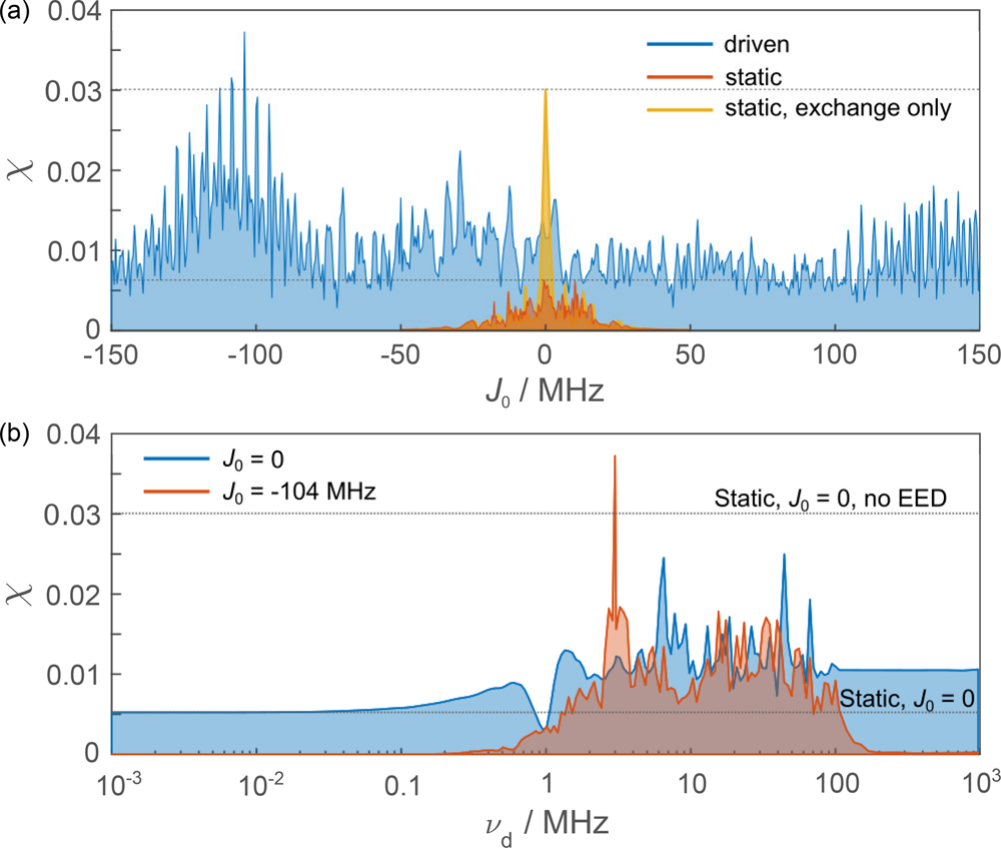
Relative anisotropy χ of models comprising 4 nuclear spins against (a) *J*_0_ for the driven system with frequency ν_d_ = 3 MHz and EED interaction *D*(*r*_0_) = −13.7 MHz, and the static case including or neglecting it, and (b) ν_d_ with *J*_0_ = 0 MHz or *J*_0_ = −104 MHz. Dotted lines represent the maximum value of static models shown in panel a at *J*_0_ = 0 MHz.

Since an enhancement in magnetosensitivity for simple driven radical pair systems comprising a single nuclear spin is observed, we now address if this MFE persists as the system is made more complex by extending it to comprise 4 nuclear spins. Specifically, we consider a flavin–tryptophan radical pair with the established driven model as before, but now include two nuclear spins for each radical with hyperfine couplings corresponding to N5 and N10, and N1 and H1, respectively. We arbitrarily choose a driving frequency of 3 MHz, found to be in the effective range for the simpler model, and an amplitude of 2 Å. Results for this model are displayed in Figure 5a, where the system in the presence of the time-dependent *J*(*t*) is compared to the static scenario for both EED and exchange interactions included, and with the hypothetical scenario with only exchange interaction.

With EED, the data resemble that of our simpler models, with enhancements up to a factor of approximately 6 as compared to the static model. For an optimal *J*_0_, the driven scenario, including both inter-radical interactions, is able to exceed the idealized static scenario for which the EED interaction is plainly neglected. As in the simpler models investigated, the MFE observed is resilient even to large exchange couplings with enhancements persisting in the range of −150 ≤ *J*_0_ ≤ 150 MHz.

In Figure 5b magnetosensitivity is shown as a function of the driving frequency for chosen values of *J*_0_; namely, *J*_0_ = 0, and *J*_0_ = −104 MHz. We find enhanced MFEs, up to a factor of 5, are realized for several frequencies in the 1–100 MHz band and *J*_0_ = 0, which generally outperforms the static scenario with EED interaction included but is less sensitive than the hypothetical static case neglecting both interactions. However, for *J*_0_ = −104 MHz our results demonstrate that the driven model with both exchange and EED interaction included, can be more magnetosensitive than the static case that neglects inter-radical interactions. Furthermore, when the EED interaction is included, which reflects what is realized in practice, the driven model outperforms the static case for the large range of *J*_0_ considered and for all driving frequencies ν_d_ ≳ 1 MHz.

## Where Do We Go Next?

We have demonstrated that a physically plausible periodic driving in the range of 1–100 MHz, which modulates the inter-radical distance of the radical pair mechanism, can overcome suppression in compass sensitivity caused by inter-radical interactions. In nature, these driving processes could be a result of internal motion, such as structural rearrangements following initial charge transfer or associated with sensory transduction.^4^ Such conformational changes of proteins following charge transfer at physiological temperatures have also been suggested as a mechanism that may mediate function in the context of proton pumping.^44^

It is pertinent to discuss the relevance of the findings here in the context of realistic systems. First, distance fluctuations on the required scale of a few angstroms are clearly observed in molecular dynamics simulations of the equilibrated radical pair state of cryptochrome.^32,36^ In more realistic models of the molecular dynamics, many modes are expected to be active and could interfere such that oscillations will lose coherence over time resulting in a motion that appears diffusive subsequent to the initial fast response. As the enhancements are predicted here for a broad range of frequencies and inter-radical couplings, we argue that protein driven motion could still provide enhancements in more complex settings provided that it comprises significant frequency components in the relevant range from 1 to 100 MHz. This assessment is further corroborated by our observations that damped oscillations can be even more effective and that, for the low-frequency modes, sustained oscillations are not a necessity as a single oscillation can already produce a significant enhancement. In the SI we have additionally considered a more realistic treatment of protein mediated radical motion with a Brownian dynamics model implemented via the stochastic Schrödinger equation^45^ (see Figure S13). This model calculation demonstrates that for weak velocity damping, the compass sensitivity can be enhanced over the static limit and even the idealized undamped harmonic model, suggesting that more realistic models could be better amplifiers. However, a comprehensive investigation of this, which is currently underway, is beyond the scope of the present study.

In the limit of diffusive motion, we expect that the effects are sustained, as LZSM transitions can be efficiently driven by diffusion through the level-anticrossing region.^46^ In fact, for fast diffusive motion, the mean efficiency of transitions between the diabatic terms is known to be strongly enhanced due to multiple passages through the intersection region. Even overdamped Brownian motion modulating the inter-radical interactions can be effective,^11^ although with smaller enhancements than realized in the present study. Hence, as motion appears to generally support the compass sense and in view of the clear enhancements induced by oscillatory motion, we anticipate that more realistic motion could be enhancing in the range of the Brownian underdamped (during initial dynamics) to Brownian overdamped (long-term dynamics) domain. In fact, the model studied in ref (32) and the model presented here correspond to the opposite limiting cases of Brownian motion in the limit of strong and weak damping, respectively. Thus, one can conclude that the protein motion present in vivo, both Markovian and non-Markovian, must be viewed as a pertinent, but as of now typically ignored, factor to realize compass sensitivity.

We note that the effects discussed here could manifest as a result of the protein environment responding to the activation processes. Such dynamics is not specific to a particular system (such as FAD^•–^/Trp^•+^ or FADH^•^/O_2_^•–^) and the radical pair mechanism, but an overarching, potentially enhancing factor. As such, the effect could likewise enable magnetosenstivity in three-radical systems, such as the proposed scavenger radical model,^28,29,31^ for which overcoming the suppressive inter-radical interaction in the immobile limit requires precise radical placement of the radical. Here, oscillatory motions could mitigate constraints on radical positions.

By maintaining relevant quantum systems in a “live” far from equilibrium state, driving may constitute a crucial addition to noise assisted processes. This could also provide an increase in robustness that potentially explains the discrepancy between in vitro experiments on isolated proteins and ethological observations of live animals.^9^ To further elucidate driving in magnetoreception and assess frequency response to protein activation, we are currently pursuing more realistic models of motion, such as system–bath models with underdamped Brownian characteristics.^47−49^ Oscillatory motion resulting from the photoactivation of cryptochrome could be identified by molecular dynamics simulations, which so far have only been used to study equilibrium configurations and for time scales insufficient to assess the desired frequency spectrum.^18,32,36^ Our initial finding using optimal quantum control suggests the scope for further enhancing sensitivity of the natural system. Future work considering an interplay of the natural processes and artificially controlled driving may provide principles for enhancements in quantum sensing and quantum inspired bioengineering, both based on the radical pair mechanism and more generally.

## Supporting Information Available

Analysis of coherence and entanglement in the single hyperfine-coupled nitrogen atom model, further discussion of general features for simplified models, demonstration of additional enhancements via quantum control, an additional result utilizing the stochastic Schrödinger equation, and results showing effect of variation in oscillation orientation/amplitude, in damped driving oscillations, and for a Brownian oscillator (PDF)
Transparent Peer Review report available (PDF)
