# Supplementary material for: Driven Radical Motion Enhances Cryptochrome Magnetoreception: Toward Live Quantum Sensing
Source: J Phys Chem Lett. 2022 Nov 4;13(45):10500–6. doi: 10.1021/acs.jpclett.2c02840 (PMC9677492; doi:10.1021/acs.jpclett.2c02840)
Supplement: Supplementary file 1 — jz2c02840_si_001.pdf [file jz2c02840_si_001.pdf]

Supporting Information

Driven Radical Motion Enhances Cryptochrome Magnetoreception: Towards Live Quantum Sensing

Luke D. Smith, Farhan T. Chowdhury, Iona Peasgood, Nahnsu Dawkins, & Daniel R. Kattnig*
Living Systems Institute and Department of Physics
University of Exeter, Stocker Road, Exeter EX4 4QD, United Kingdom
E-mail: d.r.kattnig@exeter.ac.uk

In this supporting material, we present further justification and analysis of claims made in the main text. Firstly, the computational details for our models are outlined. Subsequently, we provide some detail and exposition for the Floquet approach of simulating time-evolution. This is followed by analysis of coherence and entanglement in the single hyperfine-coupled nitrogen atom model. We then consider this model under further simplifications of the zero perpendicular hyperfine coupling component, and time averaged recombination and exchange, and show that general features persist for a simple two-level system. Next, we look at damped driving oscillations and demonstrate enhancements for a simple radical pair model driven externally with optimal quantum control, which exceeds enhancements due to harmonic driving shown in our work. We lastly consider the effect of variation in oscillation orientation and amplitude, and also consider results obtained with the stochastic Schrödinger equation.

COMPUTATIONAL AND MODEL DETAILS

The following open source packages were used: NumPy [1]; SciPy [2]; QuTiP [3]; Matplotlib [4].

**Nucleus**	Hyperfine interaction tensor (mT)
N5	$\begin{bmatrix} -0.0995 & 0.0029 & 0 \\ 0.0029 & -0.0875 & 0 \\ 0 & 0 & 1.7569 \end{bmatrix}$
N10	$\begin{bmatrix} -0.0149 & 0.0021 & 0 \\ 0.0021 & -0.0237 & 0 \\ 0 & 0 & 0.6046 \end{bmatrix}$

TABLE I. Hyperfine coupling parameters for nuclei in the flavin radical in mT.

**Nucleus**	Hyperfine interaction tensor (mT)
N1	$\begin{bmatrix} -0.0337 & 0.0924 & -0.1353 \\ 0.0924 & 0.3303 & -0.5318 \\ -0.1353 & -0.5318 & 0.6679 \end{bmatrix}$
H1	$\begin{bmatrix} -0.9921 & -0.2091 & -0.2003 \\ -0.2091 & -0.2631 & 0.2803 \\ -0.2003 & 0.2803 & -0.5398 \end{bmatrix}$

TABLE II. Hyperfine coupling parameters for nuclei in the tryptophan radical in mT.

For the main text we use hyperfine-coupling parameters[1] corresponding to the nitrogen atom N5 in flavin

[1] For the single hyperfine interaction model.

and additionally N10 in flavin, and N1 and H1 for tryptophan used in the more complex model study. The values for these parameters in mT are shown in Table I for flavin and Table II for tryptophan. Furthermore, for all simulations we assume a geomagnetic field strength of $B_0 = 50\,\mu$T, reaction rate constants $k_{b_0} = 2\,\mu$s and $k_f = 1\,\mu$s, range parameter $\beta = 1.4\,\text{Å}^{-1}$. For simulation of relative anisotropy we compute dynamics up to a maximum time of $t = 12.5\,\mu$s for which the reaction dynamics are complete. Position of the tryptophan relative to flavin is given by [8.51, -14.25, 6.55] Å. The hyperfine interactions of electron spins $\hat{\mathbf{S}}_i$ in each radical i with n_i nuclear spins $\hat{\mathbf{I}}_{i,j}$, for $j \le n_i$, is described by the hyperfine interaction Hamiltonian

$$\hat{H}_{\text{hf}} = \sum_{i=1}^{m} \hat{\mathbf{S}}_i \cdot \left(\sum_{j=1}^{n_i} \mathbf{A}_{i,j} \cdot \hat{\mathbf{I}}_{i,j} \right), \tag{S1}$$

where $\mathbf{A}_{i,j}$ is the hyperfine coupling tensor for nuclear spin j in radical i. The Zeeman interaction of the electron spins with the external magnetic field $\mathbf{B}$ is described by $\hat{H}_{\text{Zee}} = -\frac{1}{\hbar} g_i \mu_B \hat{\mathbf{S}}_i \cdot \mathbf{B}$, where μ_B is the Bohr magneton and g_i is the g-value of radical i. The exchange interaction of the electron spins is described by $\hat{H}_{\text{ex}}(t) = -2J(t)\hat{\mathbf{S}}_i \cdot \hat{\mathbf{S}}_j$, where the strength $J(t)$ is dependent on the inter-radical distance modulation described in the main text. Magnetic electron-electron dipolar interactions under the point dipole approximation are accounted for in the Hamiltonian

$$\hat{H}_{\text{dip}}(t) = -\sum_{i>j}^{m} D_{ij}(|r_{ij}(t)|) \Big(3(\hat{\mathbf{S}}_i \cdot \mathbf{u}_{ij})(\hat{\mathbf{S}}_j \cdot \mathbf{u}_{ij}) - (\hat{\mathbf{S}}_i \cdot \hat{\mathbf{S}}_j) \Big), \tag{S2}$$

where $D_{ij}(\mathbf{r}_{ij}(t)) = \mu_0 g_i g_j \mu_B^2 / (4\pi\hbar |\mathbf{r}_{ij}(t)|^3)$, for which μ_0 is the permeability of free space and $\mathbf{r}_{ij}(t)$ defines the time-dependent displacement between radicals such that $\mathbf{u}_{ij} = \mathbf{r}_{ij}(t)/|\mathbf{r}_{ij}(t)|$ is the unit vector

FLOQUET THEORY

Floquet theory is well suited to the study of strongly driven periodic quantum systems. By essentially transforming the problem of solving a Hamiltonian with a complex time dependence (potentially involving many Fourier components) into a time-independent problem [5], the approach lends itself to both increased computational efficiency and scalability. One way it allows for this is by intrinsically evading secular terms (terms not periodic in the time variable [6]). Variants of Floquet theory have seen uses in fields ranging from solid state nuclear magnetic resonance to multiphoton spectroscopy, but its application to radical pair recombination reactions under time-dependent magnetic fields was first shown in Ref. 7.

The theory is frequently applied for quantum dynamics under unitary evolution, but also generalises for the non-unitary evolution [8, 9] induced by H_{eff}. Specifically, the propagator can be expressed as

$$U(t, 0) = \phi(t) e^{-iEt} V^{-1}, \tag{S3}$$

where $\phi(t)$ is the matrix of Floquet modes and the diagonal matrix of quasienergies E and $V = \phi(0)$ are obtained from the eigen-decomposition of the single-period propagator

$$U(T, 0) = V e^{-iEt} V^{-1} \tag{S4}$$

The Floquet modes are periodic in t, i.e. $\phi(t) = \phi(t + T)$, and can be obtained from $\phi(t) = U(t, 0) V e^{iEt}$. So the propagator can be constructed for arbitrarily large times by integrating Eq. (S3) over simply two periods: once to evaluate the one-period propagator, from which $\phi(0)$ and the quasienergies are obtained, and once more subsequently to evaluate $\phi(t)$ at the required times. Due to the periodicity of $\phi(t)$, the latter only necessitates the evaluation for $t \bmod T \in [0, T]$.

ANALYSIS OF COHERENCE AND ENTANGLEMENT

Several different measures of entanglement and coherence were used to probe the radical pair mechanism in previous studies. Here, we outline a selection of these additional measures to provide further analysis of coherence and entanglement in our driven radical pair model. We first address the results of coherence and entanglement measures, which are typically defined with respect to a normalised density matrix $\hat{\rho}$ and are in general basis dependent. For instance, the relative entropy of coherence utilised in the main text,

$$\mathcal{C}_r[\hat{\rho}] = S[\mathbb{IC}(\hat{\rho})] - S[\hat{\rho}], \tag{S5}$$

is defined with respect to a basis $\{|n\rangle\}_{n=1}^{d}$ of the d-dimensional Hilbert space. Here, $S[\hat{\rho}] = -\mathrm{Tr}[\hat{\rho}\log(\hat{\rho})]$ represents the von Neumann entropy and $\mathbb{IC}(\hat{\rho}) = \sum_n |n\rangle\langle n|\hat{\rho}|n\rangle\langle n|$ is a dephasing operation that returns a density matrix with its off-diagonal terms eliminated. Another commonly used coherence measure, the l_1-norm, is defined as

$$\mathcal{C}_{l_1}[\hat{\rho}] = \sum_{nm} |\langle n|\hat{\rho} - \mathbb{IC}(\hat{\rho})|m\rangle| = \sum_{n\neq m} |\langle n|\hat{\rho}|m\rangle|, \tag{S6}$$

and simply sums over the moduli of the off-diagonal terms in a given density matrix. Furthermore, Kominis defined a coherence measure [10] based on the relative entropy

$$\mathcal{C}_{st}[\hat{\rho}] = S[\hat{P}_S\hat{\rho}\hat{P}_S + \hat{P}_T\hat{\rho}\hat{P}_T] - S[\hat{\rho}], \tag{S7}$$

which alters the dephasing operation in the relative entropy of coherence to singlet and triplet projection operators $\hat{P}_S$ and $\hat{P}_T$, respectively. This measure aims to report singlet-triplet coherence while being independent of basis and unaffected by the coherence between triplet states. A similar measure of singlet-triplet coherence was used in Ref. 11, but is instead based on the l_1-norm measure.

Additionally, the relative entropy of coherence with respect to the maximally mixed state is defined as

$$\mathcal{C}_1 = S\left[\hat{\rho}||\hat{1}/d\right] = \log d - S\left[\hat{\rho}\right], \tag{S8}$$

to quantify the amount of basis-independent coherence [12]. These measures are often used with respect to the electronic subspace of the full density matrix $\hat{\rho}$ by performing a trace over the nuclear spin degrees of freedom $\mathrm{Tr}_{\mathrm{nuc}}(\hat{\rho}) = \hat{\sigma}$, where $\hat{\sigma}$ represents the density matrix of electron spins. In studies concerning the electron spin coherence with basis dependent measures, it is common to choose a basis consisting of spin up/down states, or of singlet/triplet states. The former provides a measure of the electron spin correlation, whereas the latter singlet and triplet states are directly related to chemical products of the radical pair reaction. Specifically, in a system of two electrons spins, there are four states in the up-down (UD) basis $|\uparrow\uparrow\rangle$, $|\downarrow\downarrow\rangle$, $|\uparrow\downarrow\rangle$ and $|\downarrow\uparrow\rangle$. Alternatively, the singlet-triplet (ST) basis states are denoted by the singlet state $|S\rangle = (|\uparrow\downarrow\rangle - |\downarrow\uparrow\rangle)/\sqrt{2}$, and the triplet states $|T_+\rangle = |\uparrow\uparrow\rangle$, $|T_-\rangle = |\downarrow\downarrow\rangle$ and $|T_0\rangle = (|\uparrow\downarrow\rangle + |\downarrow\uparrow\rangle)/\sqrt{2}$. We make use of both basis representations to elucidate specific features with respect to driving.

As the density matrix of the radical pair system is time-dependent, so too are measures of coherence thus far discussed. However, to analyze coherence as the exchange interaction J_0 and driving frequency ν_{d} are altered, we integrate the measure over a time period relevant to the radical pair reaction. For a measure $\mathcal{C}[\hat{\sigma}(t)]$ the time-integrated coherence is given by

$$\mathcal{C} = \int_0^\infty \mathcal{C}[\hat{\sigma}(t)]\mathrm{Tr}[\hat{\sigma}(t)]\mathrm{dt}, \tag{S9}$$

which has been weighted according to the population remaining in the system using $\mathrm{Tr}[\hat{\sigma}(t)]$. In practical realisations we fix the upper limit of the time interval to $5\,\mu$s, corresponding to the time required for reaction dynamics and coherent interconversion to be complete within our model. Considerations of coherence in the radical pair

mechanism also depend on the orientation dependence with respect to the magnetic field. To address this we take an average of the coherence measure

$$\overline{\mathcal{C}} = \frac{[\mathcal{C}_{\parallel} + \mathcal{C}_{\perp}]}{2}, \tag{S10}$$

where $\mathcal{C}_{\parallel}$ and $\mathcal{C}_{\perp}$ are the time-integrated coherences of the system with respect to parallel and perpendicular orientation to the magnetic field, respectively. To evaluate $\mathcal{C}_{\parallel}$ and $\mathcal{C}_{\perp}$, the time-integrated coherence of Eq. (S9) must be calculated for the density matrix evolution with respect to the parallel $\hat{\sigma}_{\parallel}(t)$ and perpendicular $\hat{\sigma}_{\perp}(t)$ orientations. This measure thus reports on the average amount of coherence that is present in the system under different orientations with respect to a magnetic field.

Average electron spin coherence with respect to orientation

It was shown in the main text that driving which enhances sensitivity, as measured by relative anisotropy χ, also stimulates coherence as measured by $\overline{\mathcal{C}_r}$ in the ST basis. We consider the same driven system (a single hyperfine-coupled nitrogen atom) in presence of exchange interaction with oscillation amplitude fixed at $\Delta_{\mathrm{d}} = 3\,\text{Å}$. The single non-zero hyperfine interaction is assumed axial, with principal components given by $A_{xx} = A_{yy} = A_{\perp} = -2.6\,\mathrm{MHz}$ and $A_{zz} = A_{\parallel} = 49.2\,\mathrm{MHz}$. The driving likewise gives rise to a time dependent recombination $k_b(t) = k_{b0} \exp\left[-\beta(r(t) - r_0)\right]$ and exchange $J(t) = J_0 \exp\left[-\beta(r(t) - r_0)\right]$ with $r(t) = \frac{\Delta_d}{2}\left[1 - \cos(2\pi\nu_d t)\right] + r_0$, with $k_{b_0} = 2\,\mu s^{-1}$, $k_f = 1\,\mu s^{-1}$ and $\beta = 1.4\,\text{Å}^{-1}$.

In Fig. S1 we present color maps for the larger set of coherence measures introduced in this section, with a variation of J_0 against ν_{d}, and evaluate measures in both the UD and ST basis where appropriate. The relative anisotropy χ has also been included in Fig. S1(a), and $\overline{\mathcal{C}_r}$ evaluated in the ST basis in Fig. S1(b), for ease of comparison. Furthermore, we present the results of $\overline{\mathcal{C}_{l_1}}$ in the ST basis and $\overline{\mathcal{C}_{st}}$ in Fig. S1(c & d) which in general show qualitatively similar features to $\overline{\mathcal{C}_r}$.

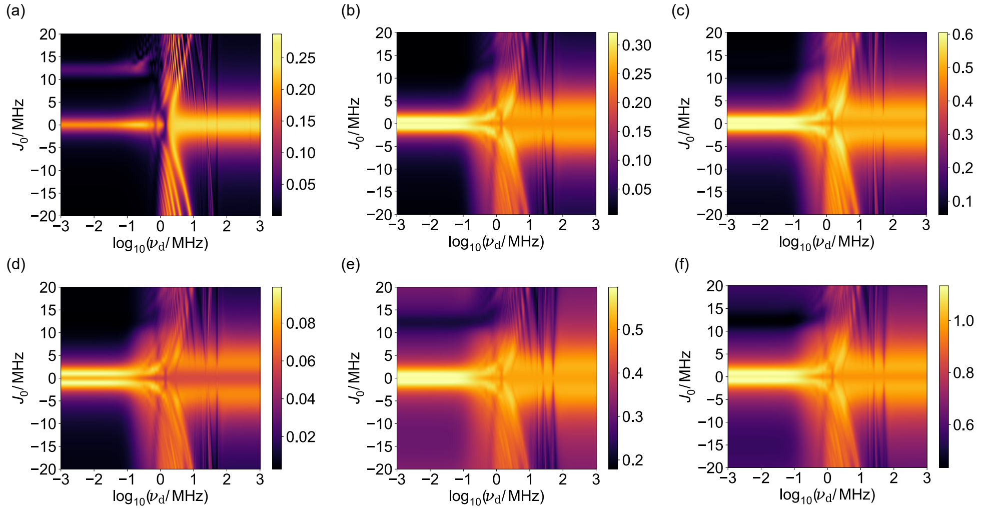

FIG. S1. Color maps of anisotropy and average of the electronic coherence measures with respect to orientation for a variation of exchange J_0 and driving frequency ν_{d} in a model driven radical pair system comprising a single hyperfine-coupled nitrogen atom. (a) Relative anisotropy χ. (b) Relative entropy of coherence $\overline{\mathcal{C}_r}$ evaluated in the ST basis. (c) l_1-norm of coherence $\overline{\mathcal{C}_{l_1}}$ evaluated in the ST basis. (d) Measure of singlet-triplet coherence $\overline{\mathcal{C}_{st}}$ (e) Relative entropy of coherence $\overline{\mathcal{C}_r}$ evaluated in the UD basis. (f) l_1-norm of coherence $\overline{\mathcal{C}_{l_1}}$ evaluated in the UD basis.

Larger differences are found for $\overline{\mathcal{C}_{st}}$, but it should be noted that this measure corresponds to ST coherence only, whereas the other measures are of coherence in the ST basis and also account for coherence between triplet

states. Therefore, a comparison of these measures provides insight into the nature of the coherence. In general, $\overline{\mathcal{C}_{st}}$ suggests that enhancements in χ are accompanied by ST coherence, whereas minor differences in $\overline{\mathcal{C}_r}$ and $\overline{\mathcal{C}_{l_1}}$ suggest that some coherence between triplet states is also present.

Further insight is gained by analysing coherence as measured by $\overline{\mathcal{C}_r}$ and $\overline{\mathcal{C}_{l_1}}$ in the UD basis, as shown in Fig. S1(e & f). In particular we observe that whilst qualitative features emerge that are in agreement with ST basis measures, there is a relatively greater amount of UD coherence for low driving frequencies. By comparing UD and ST basis states, we conclude that the coherence for this parameter range is most likely due to coherence between $|\uparrow\downarrow\rangle$ and $|\downarrow\uparrow\rangle$ states, suggesting the system is trapped in the $|S\rangle$ state. However, an increase in χ in the approximate range of $10 \lesssim J_0 \lesssim 15\,\text{MHz}$ suggests that some interconversion of singlet and triplet populations occur in this range. This provides an interpretation that the interconversion is small or does not persist over time due to the lack of ST coherence and coherence between triplet states, whilst the lack of UD coherence indicates population transfer to $|T_+\rangle$ or $|T_-\rangle$ states.

Average electron spin entanglement with respect to orientation

To analyze these features further we employ entanglement measures, such as the logarithmic negativity

$$E_N[\hat{\rho}] = \log_2 ||\hat{\rho}^{\Gamma_A}||_1, \tag{S11}$$

where $\hat{\rho}^{\Gamma_A}$ is the partial transpose of $\hat{\rho}$ with respect to a subsystem A and $||\hat{A}||_1 = \text{Tr}|\hat{A}| = \text{Tr}\sqrt{\hat{A}^\dagger\hat{A}}$ represents the trace norm of an operator $\hat{A}$. Additionally, we compute the entanglement concurrence

$$E_C[\hat{\rho}] = \max(0, \lambda_1 - \lambda_2 - \lambda_3 - \lambda_4), \tag{S12}$$

where λ_i are the eigenvalues in decreasing order of

$$R = \sqrt{\sqrt{\hat{\rho}}\tilde{\rho}\sqrt{\hat{\rho}}}, \tag{S13}$$

in which $\tilde{\rho} = (\sigma_y \otimes \sigma_y)\rho^*(\sigma_y \otimes \sigma_y)$ and σ_y is a Pauli spin matrix. As in the case of the coherence measures discussed, we evaluate the entanglement measures for the electron spin density matrix $\hat{\sigma}(t)$ and integrate over time as in Eq. (S9). Likewise, as in Eq. (S10), we take an average over measures evaluated with respect to the system with perpendicular and parallel orientations to the magnetic field to give

$$\overline{E} = \frac{[E_\parallel + E_\perp]}{2}. \tag{S14}$$

Note that unlike most of the coherence measures introduced, entanglement measures are independent of choice of basis. Thus, we have grouped together the basis-independent coherence of Eq. (S8) and these entanglement measures and presented color maps of the results in Fig. S2 for a variation of J_0 against ν_d.

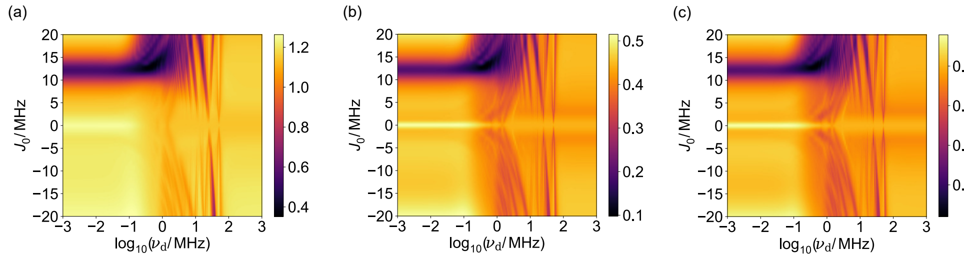

FIG. S2. Color maps of the average of electronic measures of entanglement and basis-independent coherence with respect to orientation for a variation of exchange J_0 against the driving frequency ν_d in a driven radical pair system comprising a single hyperfine-coupled nitrogen atom. (a) Basis-independent coherence $\mathcal{C}_1$ (b) Entanglement concurrence $\overline{E_C}$ (c) Logarithmic negativity entanglement $\overline{E_N}$.

Qualitatively similar features emerge in all of these measures, with generally large entanglement/basis-independent coherence present across the range of parameter choices. For, low driving frequencies and large J_0, this agrees well with our interpretation that the system is trapped in the $|S\rangle$ state, as it corresponds to a maximally entangled state. Other parameter choices that give rise to large entanglement, may also involve the maximally entangled triplet state $|T_0\rangle$. Enhancements in χ for driving frequencies in the range $0 \lesssim \nu_\mathrm{d} \lesssim 10\,\mathrm{MHz}$ are generally accompanied by a slight reduction in entanglement suggesting that it is also important for the system to evolve to $|T_\pm\rangle$ states. Lastly, the enhancement of χ at low driving frequencies in the approximate range of $10 \lesssim J_0 \lesssim 15\,\mathrm{MHz}$ is accompanied by a large relative decrease in the entanglement. This further supports our interpretation based on coherence, that the system evolution for this parameter range involves a population transfer to either the $|T_+\rangle$ or $|T_-\rangle$ state, whilst maintaining relatively lower population in the $|S\rangle$ and $|T_0\rangle$ states.

Difference of electron spin coherence with respect to orientation

In addition to taking the average of measures over parallel and perpendicular orientations of the magnetic field as in Eq. (S10), we have defined the difference of coherence as $\Delta\mathcal{C} = \mathcal{C}_\perp - \mathcal{C}_\parallel$ and the difference of entanglement as $\Delta E = E_\perp - E_\parallel$ with respect to parallel and perpendicular orientations. This treatment aims to identify if there is an associated increase in coherence or entanglement for a given orientation and is similar in its form to the definition of anisotropy. In Fig. S3 we plot color maps of the difference of coherence for a variation of J_0 against ν_d, using the same measures that were used for evaluating $\overline{\mathcal{C}}$. Features of the color maps largely coincide, suggesting that for parameters associated with enhancements, larger coherence is produced for the perpendicular orientation. It is also shown that there is relatively small difference in the UD coherence at low driving frequencies and large J_0 in contrast to the average of the UD coherence for this parameter range. This confirms the system is trapped in the $|S\rangle$ state and is freed by specific driving frequencies that also stimulates coherence predominantly in the $\perp$ system orientation.

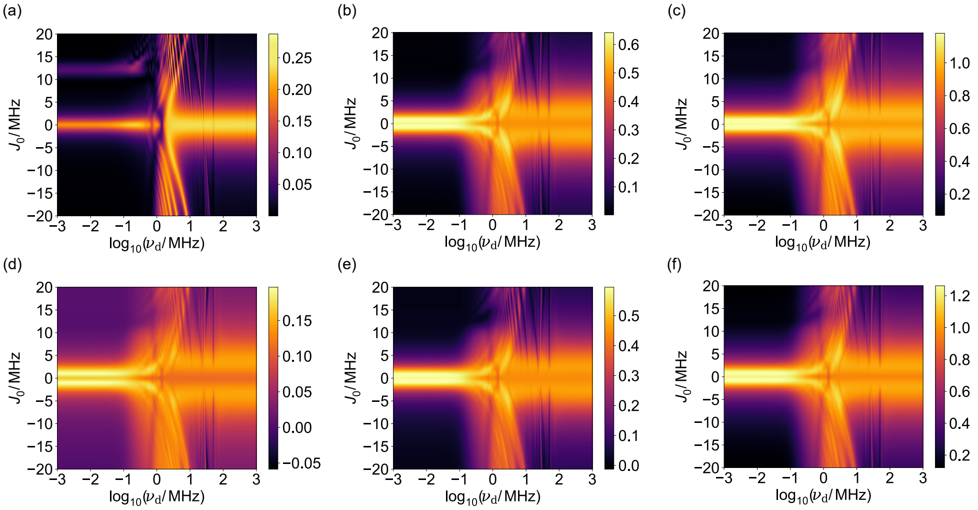

FIG. S3. Color maps of anisotropy and difference of the electronic coherence measures with respect to orientation for a variation of exchange J_0 and driving frequency ν_d in a model driven radical pair system comprising a single hyperfine-coupled nitrogen atom. (a) Relative anisotropy χ. (b) Relative entropy of coherence $\overline{\mathcal{C}_r}$ evaluated in the ST basis. (c) l_1-norm of coherence $\overline{\mathcal{C}_{l_1}}$ evaluated in the ST basis. (d) Measure of singlet-triplet coherence $\overline{\mathcal{C}_{st}}$ (e) Relative entropy of coherence $\overline{\mathcal{C}_r}$ evaluated in the UD basis. (f) l_1-norm of coherence $\overline{\mathcal{C}_{l_1}}$ evaluated in the UD basis.

Difference of electron spin entanglement with respect to orientation

Results of difference of coherence for the basis-independent measure of Eq. (S8) is shown in Fig. S4(a), whilst difference of entanglement is shown in Fig. S4(b & c). As was the case for the average, similar features are seen

amongst different measures. In general, for low driving frequencies, the difference is close to zero, indicating the system is in the $|S\rangle$ state for both orientations. However, for $10 \lesssim J_0 \lesssim 15\,\mathrm{MHz}$ a small change is observed with larger entanglement in the perpendicular orientation, suggesting that reduction in entanglement in the parallel orientation is due to redistribution of population to the $|T_\pm\rangle$ states. Lastly, we observe that for parameter ranges

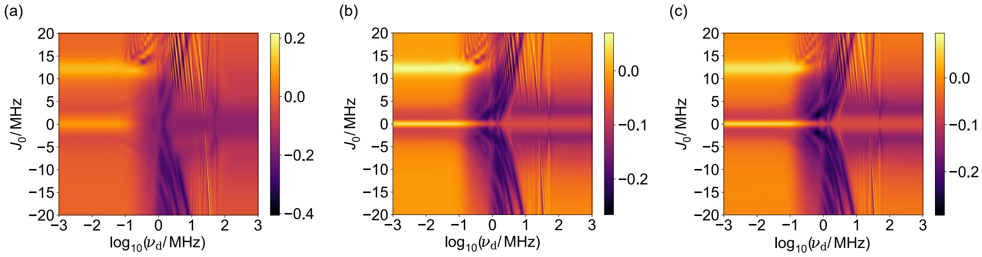

FIG. S4. Color maps of the difference of electronic measures of entanglement and basis-independent coherence with respect to orientation for a variation of exchange J_0 against the driving frequency ν_d in a driven radical pair system comprising a single hyperfine-coupled nitrogen atom. (a) Basis-independent coherence $\overline{\mathcal{C}_1}$ (b) Entanglement concurrence $\overline{E_C}$ (c) Logarithmic negativity entanglement $\overline{E_N}$.

associated with enhancements the entanglement in the perpendicular orientation is lower than in the parallel. This supports our coherence motivated interpretation that some redistribution to $|T_\pm\rangle$ accompanies the enhancement in the range of $0 \lesssim \nu_\mathrm{d} \lesssim 10\,\mathrm{MHz}$.

Electron and nuclear spin global coherence

In addition to the electron spin measures of coherence and entanglement we have considered, it is possible to compute a measure of the global coherence of the combined electron and nuclear system, i.e, of $\hat{\rho}$. We have computed this for the l_1 norm of coherence, as before time-integrating as in Eq. (S9), and taking the average or difference over orientations with results shown in Fig. S5(a & b). In Fig. S5(c) we show a global measure of coherence defined by Cai and Plenio [13] which constitutes the field-independent ($B = 0$) singlet recombination yield due to the coherent part of the initial density operator $\mathbb{GC}(\hat{\rho}(0))$, with $\mathbb{GC}(\hat{\rho}) = \hat{\rho} - \mathbb{IC}(\hat{\rho})$ evaluated in the eigenbasis of the hyperfine Hamiltonian:

$$[\mathcal{C}_y^{\mathcal{G}}]_{B=0} = \left| Y_S\left(\hat{\rho}(0) = (z_1 z_2)^{-1}\mathbb{GC}(\hat{P}_S); B = 0\right)\right|. \tag{S15}$$

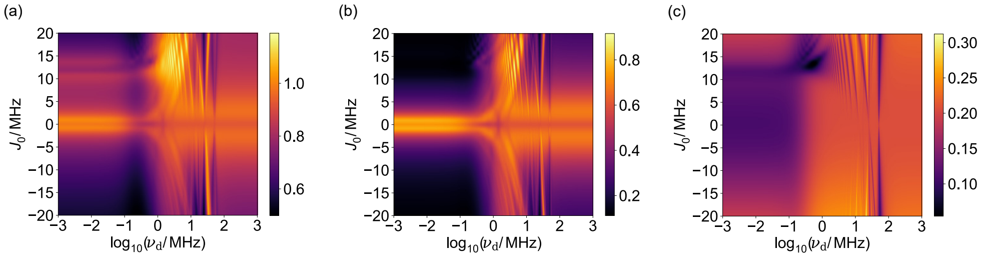

FIG. S5. Color maps of the global coherence for a variation of exchange J_0 against the driving frequency ν_d in a model driven radical pair system comprising a single hyperfine-coupled nitrogen atom. (a) The average of global coherence using $\mathcal{C}_{l_1}$ with respect to orientations (b) The difference of global coherence using $\mathcal{C}_{l_1}$ with respect to orientations. (c) Global coherence measured by the coherent contribution to singlet yield $[\mathcal{C}_y^{\mathcal{G}}]_{B=0}$.

In general the global coherence features do not coincide as strongly with the relative anisotropy χ enhancements for the simple model considered. However, certain parameter ranges such as $10 \lesssim J_0 \lesssim 20\,\mathrm{MHz}$ and $0 \lesssim \nu_\mathrm{d} \lesssim 10\,\mathrm{MHz}$, suggest that global coherence as measured by $\mathcal{C}_{l_1}$ may play a role in the spin dynamics of specific enhancements. In contrast, features of global coherence as measured by $[\mathcal{C}_y^{\mathcal{G}}]_{B=0}$ are less intricate and show the global coherence increases for driving frequencies above $\nu_\mathrm{d} \approx 1\,\mathrm{MHz}$, with further increases observed at large J_0.

Time-resolved coherence and entanglement

We have thus far time-integrated the coherence and entanglement to analyze different parameter choices of J_0 and ν_d. Here, to understand the dynamical properties of driving on entanglement and coherence we analyze the measures as a function of time. Live quantum effects have previously been considered in the context of dynamical entanglement by Cai *et al* [14], who demonstrated that entanglement can persistently recur in a simple two-spin system that is periodically driven and coupled to a noisy environment.

Assuming that the spin coupling and the energy gap of the system are modulated by the classical motion, the authors have demonstrated the cyclic generation of fresh entanglement, even under conditions for which the static thermal state is separable for all possible spin-pair configurations. For the one-nitrogen radical pair model introduced in the main text, and a parameter choice of driving oscillation amplitude $\Delta_\mathrm{d} = 3\,\text{Å}$, exchange interaction strength $J_0 = 20\,\mathrm{MHz}$, and driving frequency $\nu_\mathrm{d} = 3.8\,\mathrm{MHz}$, we also observe that the driving motion periodically boosts the electronic entanglement. This entanglement rejuvenation is evident in the singlet probability of the survived radical pairs as well as the concurrence of the electronic spin $E_C[\hat{\sigma}(t)]$, as is shown in Fig. S6(a). Both measures peak as the radical pairs are driven to small inter-radical distances, i.e. when the inter-radical coupling

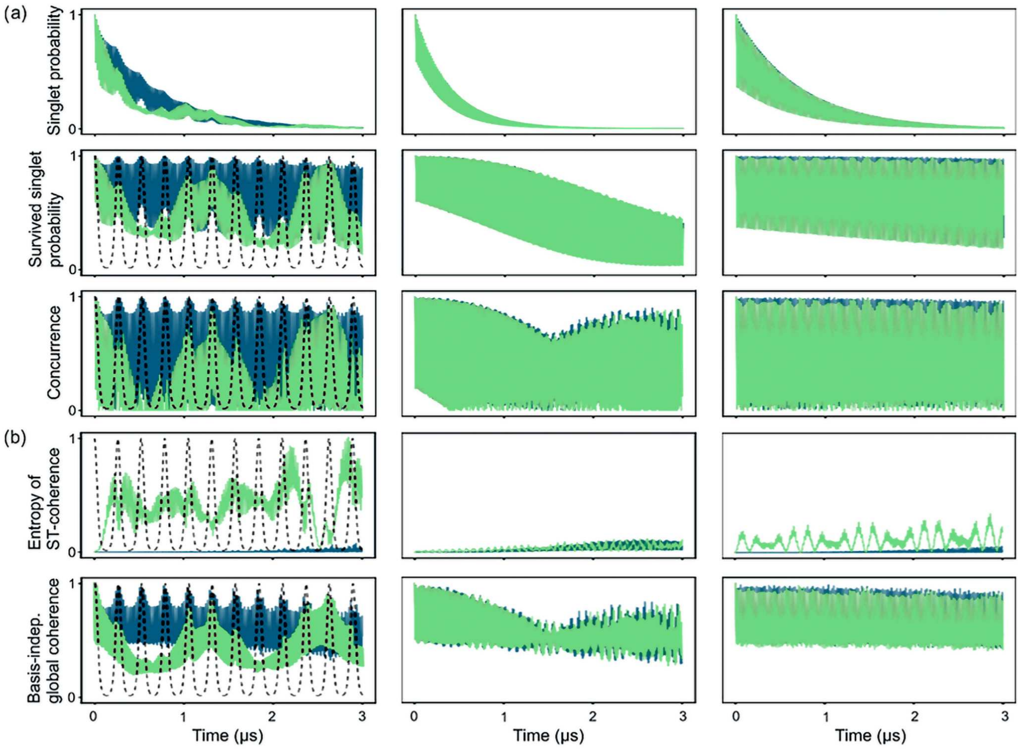

FIG. S6. In (a), we show singlet probability, survived singlet probability, and entanglement concurrency for a one-nitrogen radical pair. Results are shown for a parallel (dark blue) or perpendicular (bright green) orientation of the principal hyperfine axis with respect to the magnetic field with a fixed driving oscillation amplitude $\Delta_\mathrm{d} = 3\,\text{Å}$, exchange interaction strength $J_0 = 20\,\mathrm{MHz}$, and driving frequency $\nu_\mathrm{d} = 3.8\,\mathrm{MHz}$. For the set of simulations displayed on the left, the exchange coupling and singlet recombination yield were time-dependent as described in our driving model. For the central column and right column of data these two parameters were constant and equal to their respective value at $t = 0$ or their time-average over one driving period $1/\nu_\mathrm{d}$, respectively. For the driven system, the dashed black lines illustrate the time-dependence of the exchange coupling or recombination rate constant normalised to 1 for ease of comparison. In (b), we present the relative entropy of coherence $\mathcal{C}_r$ evaluated in the ST basis, and basis-independent coherence of the one-nitrogen radical pair. For the central and right columns of data the exchange and recombination rate were constant and equal to their respective value at $t = 0$ or their time-average over one driving period $1/\nu_\mathrm{d}$, respectively. For the driven system, the dashed black lines illustrate the time-dependence of the exchange coupling or normalised recombination rate constant.

is engaged. Note however that the effect is present for both canonical orientations of the static magnetic field, suggesting that entanglement rejuvenation does not directly promote high compass fidelity. Figure S6(b) shows that large compass sensitivity of the driven systems concurs with the generation of large singlet-triplet electronic coherence as assessed via $\mathcal{C}_r[\hat{\sigma(t)}]$ evaluated in the ST basis and a large reduction of global coherence (as assessed by a basis-independent coherence measure) for the perpendicular magnetic field direction. We further show that for zeroed perpendicular hyperfine couplings or a time-averaged recombination rate the orientation dependence is largely removed. We consider these system simplifications in more detail in the next section for the recombination yields and associated compass fidelities (see Fig. S7).

FURTHER SIMPLIFICATIONS OF THE DRIVEN RADICAL PAIR MODEL

Using the same inter-radical distance driving as described in the main text, we first consider the case of a single hyperfine-coupled nitrogen atom (I = 1) in one radical and no hyperfine interactions in the other. Likewise, the single non-zero hyperfine interaction has been assumed axial, with principal components given by $A_{xx} = A_{yy} = A_\perp = -2.6\,\text{MHz}$ and $A_{zz} = A_\parallel = 49.2\,\text{MHz}$, and reaction rates $k_{b_0} = 2\,\mu s^{-1}$, and $k_f = 1\,\mu s^{-1}$. An arbitrary driving frequency of $\nu_\text{d} = 3\,\text{MHz}$, within the identified effective range, and oscillation amplitude of $\Delta_\text{d} = 2\,\text{Å}$ is chosen. For modulation of the inter-radical coupling strength J_0, Fig. S7(a) shows recombination yields $\Phi_\parallel$, and $\Phi_\perp$ for a static magnetic field pointing in parallel and perpendicular directions, respectively, and the relative anisotropy χ defined as $|\Phi_\parallel - \Phi_\perp|/\max(\Phi_\parallel, \Phi_\perp)$. We seek to reduce complexity of this model while retaining essential features, like resilience to inter-radical coupling, to further understand the nature of the effect. To that end, we first observe that the effect is not crucially dependent on the non-zero perpendicular hyperfine coupling components. While it is true that the magnetic field effect (MFE) is more complex for $A_\perp \neq 0$, the main features are retained for $A_\perp = 0$, as shown in Fig. S7(b). Second, we note that the effect is not critically, i.e. as concerning qualitative features, dependent on the time-dependence of the recombination processes, which in essence corresponds to a stroboscopic observation of singlet state dynamics. Specifically, substituting the time-dependent $k_b(t)$ by its average over integer multiples of the driving period, we obtain results as shown in Fig. S7(c), where the same qualitative picture emerges as for Fig. S7(b). The time average was calculated as

$$\left\langle e^{-\beta\,\Delta(r(t)-r_0)}\right\rangle = e^{-\frac{\beta\,\Delta_d}{2}} I_0\left(\frac{\beta\,\Delta_d}{2}\right), \tag{S16}$$

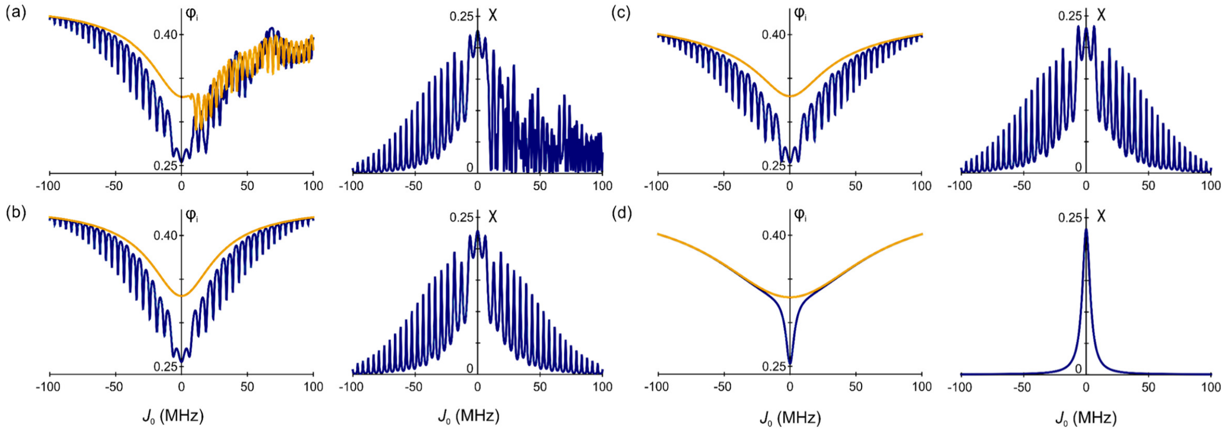

FIG. S7. Recombination yields and associated compass fidelities for a one-nitrogen radical pair with axial hyperfine interaction (principal component $A_\parallel = 49.2\,\text{MHz}$ and scalar inter-radical coupling, $\hat{H}_{12} = -2J\,\hat{\mathbf{S}}_1 \cdot \hat{\mathbf{S}}_2$). For (a) with $A_\perp = -2.6$ MHz, $J(t)$ and the singlet recombination rate $k_b(t)$ are time-dependent, whereas for (b)-(d) $A_\perp = 0$. For (c) recombination rate $k_b(t)$ is replaced by the time-average over one oscillation period. Finally, for d) the time-averaged quantities were used in place of both $J(t)$ and $k_b(t)$, i.e. the effective Hamiltonian is time-independent.

where the range parameter $\beta = 1.4\,\text{Å}^{-1}$ and I_0 denotes the modified Bessel function of first order $n = 0$.

However, if we also average the time-dependence of the exchange coupling using Eq. (S16), the enhancements are diminished and we reproduce the static radical pair mechanism result of MFE suppression due to large inter-radical interactions (see Fig. S7(d)). Thus, we conclude that the effect of driving inter-radical distance, as observed here, is predominantly due to the time-dependence of the inter-radical coupling and not dependent on the non-zero $A_\perp$ or the time-dependence of the recombination process. Note however that if the non-zero $A_\perp$ was retained, an analogous result to Fig. S7(d) would have been obtained except for a second peak of magnetosensitivity at ≈ -12 MHz that emerges in the static limit as a consequence of a level crossing (i.e. for $A_\perp = 0$; level-anti-crossing for $A_\perp \neq 0$; in zeroth order, for the magnetic field parallel to $A_\parallel$ the crossing occurs at $J = \frac{1}{4}(2\omega_0 - A_\parallel)$between $|T_+, m_I = -1\rangle$ and $|S, m_I = 0\rangle$). In the main text, the Hamiltonian for the magnetic field pointing along the perpendicular direction was shown for $A_\perp = 0$. We now further analyze the structure of the Hamiltonian with $A_\perp = 0$ for a static magnetic field applied in the parallel direction. As the Hamiltonian commutes with the z-component of the nuclear spin, $\hat{I}_z$, for both directions of the applied field, the magnetic quantum number of the nitrogen atom $m_I \in \{1, 0, -1\}$ can be used to label the energy eigenstates. For the field pointing along the parallel z-axis, with basis states chosen as $|T_+\rangle$, $|T_0\rangle$, $|T_-\rangle$ and $|S\rangle$, the m_I-sector of the Hamiltonian is given as

$$\hat{H}_\parallel = \begin{pmatrix} am_I + \omega_0 - J & 0 & 0 & 0 \\ 0 & -J & 0 & am_I \\ 0 & 0 & -am_I - \omega_0 - J & 0 \\ 0 & am_I & 0 & J \end{pmatrix}, \tag{S17}$$

where $a = A_\parallel/2$ is introduced for succinctness. Similarly, for static magnetic field applied along the x-axis

$$\hat{H}_\perp = \begin{pmatrix} am_I - J & b & 0 & 0 \\ b & -J & b & am_I \\ 0 & b & -am_I - J & 0 \\ 0 & am_I & 0 & J \end{pmatrix}, \tag{S18}$$

where $b = \omega_0/\sqrt{2}$. We have already described how the inclusion of driving introduces a Landau-Zener type transition that mediates transitions between the $|S\rangle$ and $|T_0\rangle$ state. Here, by comparing $\hat{H}_\perp$ and $\hat{H}_\parallel$, it is revealed that $\hat{H}_\perp$ allows further evolution to $|T_\pm\rangle$ states, whereas no additional coherent interconversion with $|T_\pm\rangle$ states is possible for $\hat{H}_\parallel$. This contributes to the magnetosensitivity of the system by enacting a different response for perpendicular and parallel magnetic field orientations. In the case of inter-radical coupling and A_{zz}

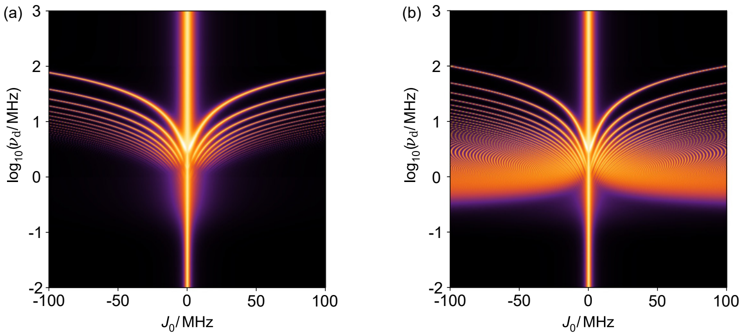

FIG. S8. Color map of state-transition efficiency, for two-level system with Hamiltonian $\hat{H}(t) = J(t)\hat{\sigma}_z + b\hat{\sigma}_x$, with constant $b = 1.4$ MHz, and $J(t)$ periodic with period $1/\nu_\text{d}$ as function of driving frequency ν_d and the maximal energy gap J_0. For (a) $J(t) = J_0 \exp[-\beta(r(t) - r_0)]$ with $r(t) = \frac{\Delta_d}{2}[1 - \cos(2\pi\nu_d t)] + r_0$ while for (b) $J(t) = J_0[1 - (r(t) - r_0)/\Delta_d]$, i.e. $J(0)$ is harmonically oscillating between J_0 and 0. We chose an oscillation amplitude of $\Delta_\text{d} = 2$ Å, and range parameter $\beta = 1.4\,\text{Å}^{-1}$ The transition efficiency is evaluated as $\int_0^\infty k \exp(-kt)|c_2(t)|^2 \text{d}t$, where $k = 1\,\mu\text{s}^{-1}$ and $|c_2(t)|^2$ is probability of finding the system in the initially unoccupied state.

that are comparable in magnitude, MFEs are small for the static system as the dynamics are characterised by fast $|S\rangle$-$|T_0\rangle$ interconversion. Consequently, there is only a marginal magnetic field response for $\hat{H}_\perp$ and $\hat{H}_\parallel$ is independent of the magnetic field. However, the inclusion of driving from molecular motion enhances directional magnetosensitivity as the periodic reduction of inter-radial coupling enables $|T_\pm\rangle$ redistribution for $\hat{H}_\perp$ (leading to reduction in singlet population), but it is unaffected for $\hat{H}_\parallel$.

For more complex systems with several hyperfine couplings, as seen in the main text, intricate magnetic field dependent spin dynamics can arise, with non-adiabatic transitions initiated by avoided-crossings that occur at non-zero J_0. For such scenarios, our description provides only a qualitative picture. However, a simpler two level system with energy-gap driven as assumed above, i.e. with $\hat{H}(t) = J(t)\hat{\sigma}_z + b\hat{\sigma}_x$ (where $\hat{\sigma}_i$ are Pauli operators), exemplifies that general features persist as complexity is increased, as is shown in Fig. (S8). Specifically, spikes in magnetosensitivity, as both driving frequencies ν_d and J_0 are modulated, are associated with the minima and maxima of the Floquet quasienergies of the system.

DAMPED DRIVING OSCILLATIONS

In the natural setting driving may be hindered by constrained protein motion or interaction with the environment leading to damped oscillations. Here, we analyze this by considering damped inter-radical distance modulation $r(t) = r_0 + \frac{\Delta_\mathrm{d}}{2}[1 - \cos(2\pi\nu_\mathrm{d}t)]\exp(-\tau^{-1}t)$, with recombination rate $k_b(t) = k_{b_0}\exp[-\beta[r(t) - r_0]]$, and $J(t) = J_0\exp[-\beta[r(t) - r_0]]$, for a choice of, $k_{b_0} = 2\,\mu$s, range parameter $\beta = 1.4\,\text{Å}^{-1}$, and oscillation amplitude of $\Delta_\mathrm{d} = 1.5\,\text{Å}$. We study the case of the single hyperfine-coupled nitrogen atom with exchange interaction introduced in the main text.

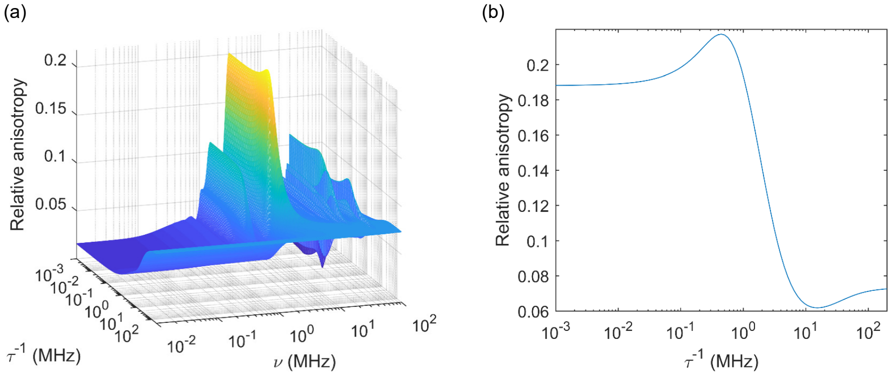

FIG. S9. Relative anisotropy χ for a choice of $J_0 = -5\,$MHz, range parameter $\beta = 1.4\,\text{Å}^{-1}$, and oscillation amplitude of $\Delta_\mathrm{d} = 1.5\,\text{Å}$. (a) Shown as a function of the inverse of the damping period τ^{-1} and driving frequency ν_d. (b) Fixed driving frequency at maximum χ as a function of τ^{-1}.

The relative anisotropy for this model is shown in Fig. S9 as a function of driving frequency ν_d and inverse of the damping period τ^{-1}. The results indicate that for oscillations that are damped too swiftly, i.e. for $\tau^{-1} \gtrsim 1\,$MHz, there is a relative decrease in the anisotropy χ as the system will begin to resemble the case of static radical pairs. For weaker damping with $\tau^{-1} \lesssim 0.1\,$MHz, the modulation of inter-radical distance persists for longer and resembles the damping free regime studied in the main text which shows relative enhancement over the static case. However, the optimal result is found for when damping and driving occur on a similar timescale to the reaction dynamics in the range of $1 - 10\,\mu$s.

SIMPLE MODEL UNDER OPTIMAL QUANTUM CONTROL

Optimal Quantum Control comprises the class of optimization algorithms which use electromagnetic "pulses" designed using a minimal time approach [15, 16] to drive a given quantum system to reach a particular target

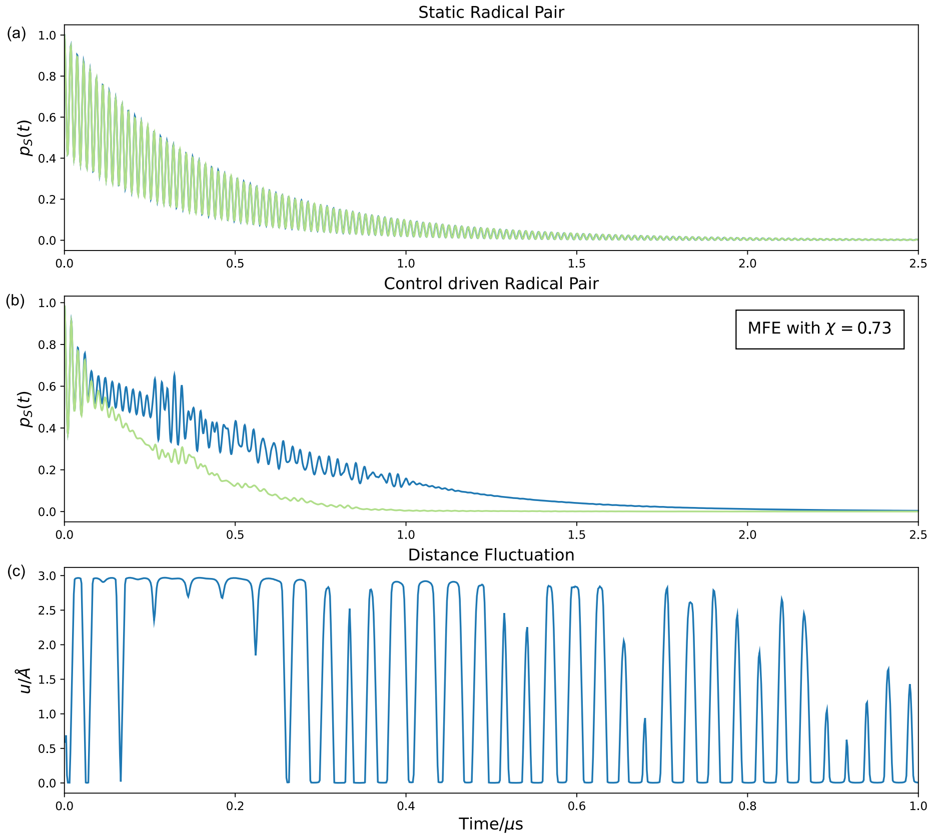

FIG. S10. Singlet yield population as a function of time for quantum control driven one nitrogen radical pair ($I = 1$) subject to distance-dependent exchange interaction and recombination. Given a time period of 10 nanoseconds and a roughly 100 MHz frequency, the maximal timescale for a transition from 3 Å to 0 Å is 1 amplitude per 5 nanoseconds. The static case is shown in (a). For control driven case (b), absolute anisotropy is minimised using optimization with 4000 time steps and 200 sampling points, with green corresponding to the the hyperfine tensor for the magnetic field along the axis $A_\perp$ and blue corresponding to the hyperfine tensor for the magnetic field along the axis $A_\parallel$. In (c), the distance fluctuation for the quantum control driven case is shown.

state. Without loss of generality, the Hamiltonian for a system undergoing quantum coherent spin dynamics can be written as:

$$\hat{H}(t) = \hat{H}_0 + \sum_{k=1}^{n} f_k(t)\hat{H}_k, \tag{S19}$$

with $\hat{H}_0$ being the drift Hamiltonian describing the time-independent part of the system, f_k are the control fields and $\hat{H}_k$ is the set of control Hamiltonians coupling the fields to the system, for example via Zeeman or dipole interactions. The values taken by controls for a system undergoing quantum coherent spin dynamics, which in our case corresponds to modulation of the inter-radical distance, can be parameterised by piece-wise constant control amplitudes in the time domain.

We apply optimal quantum control in this sense to a single hyperfine-coupled nitrogen radical pair, with principal components given by $A_{xx} = A_{yy} = A_\perp = -2.6$ MHz and $A_{zz} = A_\parallel = 49.2$ MHz, $k_{b_0} = 2\,\mu s^{-1}$, $J_0 = 10$ MHz, $k_f = 1\,\mu s^{-1}$. A “speed limit” of 3 Å is imposed to ensure that the inter-radical distance does not fluctuate at unrealistic speeds. Using pulses to externally drive spin dynamics in the radical pair, we induce hyperfine interaction mediated transitions between $|S\rangle/|T_\pm\rangle$ states. Since we have what boils down to essentially a bilinear control problem, the optimal solution jumps between two boundaries, and so control of the *bang-bang* type is optimal, with a single control function $f(t)$ and states reaching their target at fixed time intervals. By

formulating a piecewise constant Hamiltonian, with total time subdivided into fixed intervals, cost functions are computed by sampling the intervals by a few time points. We optimize the escape yield, with maximal yield subject to the speed limit, using a GRAPE [17] based approach with standard Python optimization packages. Using this method, we obtain well optimized distance fluctuations with a large magnetic field effect, as seen in Fig. S10. This demonstrates how optimal quantum control can enhance the magnetic field effect in a radical pair reaction, and the enhancement obtained by this (artificial) external driving considerably exceeds what is achievable via the harmonically driven natural systems considered elsewhere in this work.

VARIATION OF DRIVING OSCILLATION ORIENTATION AND AMPLITUDE

In the main text we demonstrated the orientation dependence of driving enhancements for a single hyperfine-coupled nitrogen atom with electron-electron dipole (EED) interactions and oscillation amplitude $\Delta_\mathrm{d} = 2\,\text{Å}$. In Fig. S11 we present further cases of $\Delta_\mathrm{d} = 4\,\text{Å}$, and $\Delta_\mathrm{d} = 6\,\text{Å}$, which also show oscillations that broadly increase the inter-radical distance are effective without necessitating exact alignment. However, inter-radical modulation is more effective if it has a closer alignment with the inter-radical axis, an observation that becomes more pronounced as oscillation amplitude is increased.

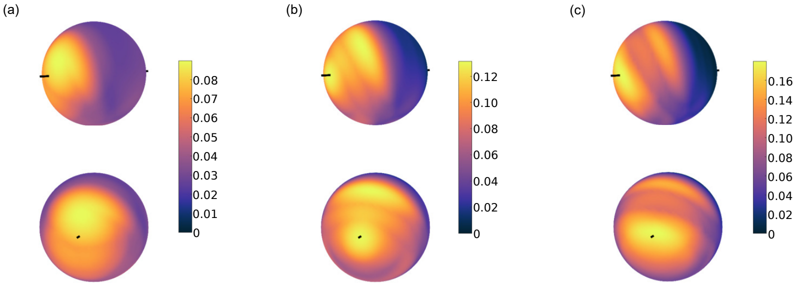

FIG. S11. Orientation dependence is displayed for a driven model comprising a single hyperfine-coupled nitrogen with EED interaction, and a fixed driving frequency of $\nu_\mathrm{d} = 4.4\,\mathrm{MHz}$. Oscillation amplitudes of (a) $\Delta_\mathrm{d} = 2\,\text{Å}$, (b) $\Delta_\mathrm{d} = 4\,\text{Å}$, and (c) $\Delta_\mathrm{d} = 6\,\text{Å}$, indicate effectiveness for broad increases of inter-radical distance.

In Fig. S12 we also consider driving that reduces the inter-radical distance rather than our standard approach of increasing it periodically. Although enhancements are more prominent for driving that increases the distance, the effect is seen to persist qualitatively for a decrease of the distance, suggesting that the time-dependent nature of the driving is also of importance.

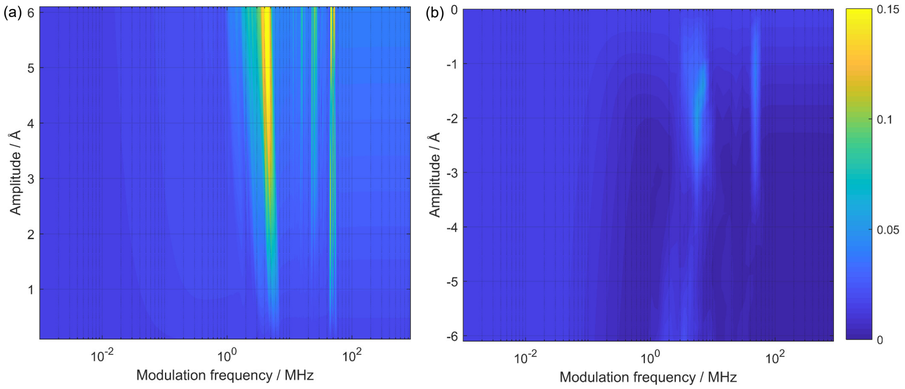

FIG. S12. Driven radical pair model with EED interactions included and $J_0 = 0$. Color map of relative anisotropy χ is shown for a variation of oscillation amplitude Δ_d against driving frequency ν_d. Driving that increases the inter-radical distance is shown on the left whilst driving that decreases it is shown on the right.

UNDERDAMPED STOCHASTIC BROWNIAN MOTION MODEL

In the main text we have considered harmonic periodic driving to elucidate the fundamental features of enhancements arising from driven radical dynamics. However, biological systems are unlikely to respond with protein conformational motion that produces radical dynamics in exactly this manner. Here we prospectively consider an arguably more realistic underdamped stochastic Brownian motion model of the radical dynamics in a biological system implemented with a stochastic Schrödinger equation.

We considered dynamics of a Brownian particle in a potential given by

$$\frac{V(x)}{m} = \frac{1}{2}\omega^2(x - x_h)^2 + \frac{A}{(x - x_b)^{12}}, \tag{S20}$$

with $\omega/2\pi = 4.9\,\text{MHz}$, $A = 1\,\mu\text{s}^{-2}\text{Å}^{14}$, $x_h = 2\,\text{Å}$, and $x_b = -2\,\text{Å}$. The second term in the potential corresponds to the repulsive part of a Lennard-Jones potential which has been added to ensure that the particle does not sample too small distances. The equations of motion are

$$\frac{\mathrm{d}}{\mathrm{d}t}x(t) = v(t) \tag{S21}$$

and

$$\frac{\mathrm{d}}{\mathrm{d}t}v(t) = -\eta v(t) - \frac{1}{m}\frac{\mathrm{d}V(x)}{\mathrm{d}x} + \xi(t), \tag{S22}$$

with a delta correlated Gaussian Langevin force $\langle \xi(t)\xi(t')\rangle = q\delta(t - t')$, where

$$q = 2\eta\frac{k_B T}{m} = 2\eta^2 D. \tag{S23}$$

We investigated the one-nitrogen radical pair model considered in the main text subject to EED interaction and $J = 0$, with a choice of $D = \omega^2 x_{th}^2/\eta$, where $x_{th} = 2\,\text{Å}$, such that $\sqrt{\langle x^2\rangle} \approx 2\,\text{Å}$ in the long time limit, thus resembling amplitudes and frequencies of motion we considered for harmonic driving. For a displacement x from initial configuration ($x = 0$ at $t = 0$), the recombination was given by $k_b = k_{b_0}\exp(-\beta x)$ with $\beta = 1.4\,\text{Å}^{-1}$, $k_{b_0} = 2\,\mu\text{s}^{-1}$ and $k_f = 1\,\mu\text{s}^{-1}$. The EED interaction is chosen such that it corresponds to that of F/WC in DmCry for $x = 0$ and was scaled for $x \neq 0$ to reflect the change of inter-radical distance whilst retaining the radicals' relative orientation. By solving the Schrödinger equation for many realisations of the noise proccess we solved for Brownian underdamped dynamics and evaluated the spread of the singlet recombination yield for

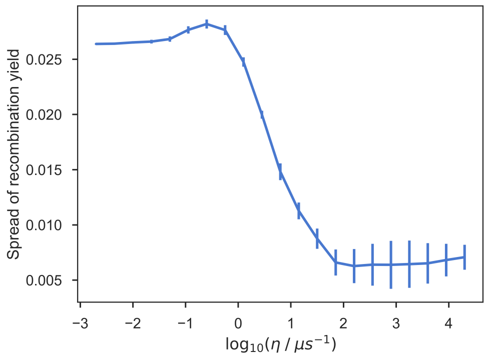

FIG. S13. Spread of the recombination yield against velocity damping constant η shown for many realisations of an underdamped stochastic Brownian motion noise process, with dynamics solved via the stochastic Schrödinger equation. For a range of up to $\eta \approx 10\,\mu\text{s}^{-1}$, there is enhancement in the spread of the recombination yield as compared to the static radical case ($\Delta_{static} = 0.0076$).

81 orientations of the magnetic field ($50\,\mu$T) on the hemisphere, where at least 3200 trajectories of $x(t)$ were sampled. The spread of the singlet yield is displayed in Fig. S13. This demonstrates that for a range of velocity damping constant up to $\eta \approx 10\,\mu\text{s}^{-1}$, the spread of the recombination yield is enhanced by approximately 3 times the static radical case ($\Delta_{static} = 0.0076$). Furthermore, weak damping appears to enhance the sensitivity beyond the undamped harmonic limit (i.e. as $\eta \to 0$). Overall the principle of enhancements found in our harmonically driven radical pair model translates to more complex and realistic considerations of radical dynamics.

CO_2-EMISSION TABLE

In Table III, we summarize climate expenses, calculated according to guidance found in [18]. The CO_2

**Numerical simulations**	
Total Kernel Hours [h]	268800
Thermal Design Power Per Kernel [W]	5.9
Total Energy Consumption [kWh]	1586
Average Emission Of CO_2 [kg/kWh]	0.47
**Were the Emissions Offset?**	Yes
Total CO_2-Emission [kg]	745

TABLE III. Carbon footprint associated with the numerical simulations.

emission per kWh used is the average global value. To try to offset the 745kg emitted, a donation was made to Atmosfair (`https://atmosfair.de`), a not-for-profit that promotes, develops and finances renewable energies in over fifteen countries worldwide.

[1] Stéfan van der Walt, S Chris Colbert, and Gael Varoquaux. The Numpy Array: a Structure for Efficient Numerical Computation. *Comp. Sci. Eng.* **2011**, *13*, 22–30.

[2] Virtanen et al. and SciPy 1. 0 Contributors. SciPy 1.0: Fundamental Algorithms for Scientific Computing in Python. *Nature Methods* **2020**, *17*, 261–272.

[3] J Robert Johansson, Paul D Nation, and Franco Nori. Qutip: An Open-Source Python Framework for the Dynamics of Open Quantum Systems. *Comp. Phys. Comm.* **2012**, *183*, 1760–1772.

[4] J. D. Hunter. Matplotlib: A 2d Graphics Environment. *Comp. Sci. Eng.* **2007**, *9*, 90–95.

[5] Jon H. Shirley. Solution of the Schrödinger Equation With a Hamiltonian Periodic in Time. *Phys. Rev.* **1965**, *138*, B979–B987.

[6] Milena Grifoni and Peter Hänggi. Driven Quantum Tunneling. *Phys. Reports* **1998**, *304*, 229–354.

[7] Hamish G. Hiscock, Daniel R. Kattnig, David E. Manolopoulos, and P. J. Hore. Floquet Theory of Radical Pairs in Radiofrequency Magnetic Fields. *J. Phys. Chem.* **2016**, *145*, 124117.

[8] P. Hänggi, et al. *Quantum Transport and Dissipation.* Wiley:Weinheim, 1998.

[9] C. Chicone. *Ordinary Differential Equations with Applications.* Springer New York: New York, 2008.

[10] I. K. Kominis. Quantum Relative Entropy Shows Singlet-Triplet Coherence is a Resource in the Radical-Pair Mechanism of Biological Magnetic Sensing. *Phys. Rev. Res.* **2020**, *2*, 023206.

[11] M. Kritsotakis and I. K. Kominis. Retrodictive Derivation of the Radical-Ion-Pair Master Equation and Monte Carlo Simulation With Single-Molecule Quantum Trajectories. *Phys. Rev. E* **2014**, *90*, 042719.

[12] Thao P. Le and Alexandra Olaya-Castro. Basis-Independent System-Environment Coherence is Necessary to Ddetect Magnetic Field Direction in an Avian-Inspired Quantum Magnetic Sensor. *arXiv:2011.15016* **2020**.

[13] Jianming Cai and Martin B Plenio. Chemical Compass Model for Avian Magnetoreception as a Quantum Coherent Device. *Phys. Rev. Lett.* **2013**, *111*, 230503.

[14] Jianming Cai, Sandu Popescu, and Hans J. Briegel. Dynamic Entanglement in Oscillating Molecules and Potential Biological Implications. *Phys. Rev. E* **2010**, *82*, 021921.

[15] Gerhard C.Hegerfeldt. Driving at the Quantum Speed Limit: Optimal Control of a Two-Level System. *Phys. Rev. Lett.* **2013**, *111*, 260501.

[16] Sebastian Deffner and Steve Campbell. Quantum Speed Limits: From Heisenberg's Uncertainty Principle to Optimal Quantum Control. *J. Phys. A* **2017**, *50*, 453001.

[17] N. Khaneja, et al. Optimal Control of Coupled Spin Dynamics: Design of NMR Pulse Sequences by Gradient Ascent Algorithms. *J. Magn. Reson.* **2005**, *172*, 296–305.

[18] Scientific CO2nduct, Raising Awareness For The Climate Impact of Science. `https://scientific-conduct.github.io` (accessed May 2022).
