# Supplementary material for: Driven Radical Motion Enhances Cryptochrome Magnetoreception: Toward Live Quantum Sensing
Source: J Phys Chem Lett. 2022 Nov 4;13(45):10500–6. doi: 10.1021/acs.jpclett.2c02840 (PMC9677492; doi:10.1021/acs.jpclett.2c02840)
Supplement: Supplementary file 2 — jz2c02840_si_002.pdf [file jz2c02840_si_002.pdf]

jz-2022-02840t.R1

Name: Peer Review Information for "Driven Radical Motion Enhances Cryptochrome Magnetoreception: Towards Live Quantum Sensing"

First Round of Reviewer Comments

Reviewer: 1

Comments to the Author

In this paper the authors present a novel and interesting idea that driven oscillations of singlet radical pair separation can enhance anisotropic magnetic field responses in the well-studied FAD-Trp radical pair which has been suggested to form the basis of avian magnetoreception. Previous studies on realistic models of this system find very low anisotropic MFE responses of the FAD-Trp pair, so new suggestions for mechanisms by which larger anisotropies can be found are clearly needed to understand the long-standing mystery of magnetoreception.

The results, which show that deterministic driving forces perturbing the inter-radical distance, even when damped, can lead to significant enhancement of anisotropic MFEs are particularly interesting, and the study is thorough.

A concern of mine is that the model of deterministic r(t) seems very physically unrealistic for a biological system. I would suggest the authors move some discussion of physical motivation for using deterministic models to where the model is introduced and include more discussion of why this driven model was chosen over arguably more realistic stochastic models for example underdamped Brownian motion (presumably computational expense of Floquet theory vs the stochastic schrodinger equation?). Ideally I would like to see at least one example (i.e. one large value of J_0 e.g. ~10MHz, perhaps with the one nuclear-spin model of FAD-Trp) of using underdamped stochastic Brownian motion for r(t), instead of the undamped harmonic model used in this paper, using the stochastic Schrodinger equation.

The authors should also clarify in their introduction what precisely they mean by "driven" in the context of their work - particularly if stochastically driven underdamped Brownian oscillator models produce similar enhancements to the undamped/deterministic cases, and clarify if/how this effect is different to previously described relaxation effects. Also it is well-established that when the FAD-Trp radical pair system is driven by radio-frequency magnetic field noise then anisotropy in the magnetic field response is suppressed, so I think it should be clarified in the title to reflect this. The title currently suggests to me that arbitrary driving can enhance sensitivity which is not the case.

The concept of a "live" magnetoreceptor is also very loose and not particularly helpful - I suggest removing discussion of this a s "live" model. If underdamped thermal Brownian motion can also produce enhanced anisotropy then I would argue that describing the enhancement effect as "live quantum sensing" is misleading.

I also believe the discussion around equation (10) explaining the origin of the enhanced anisotropic needs further expansion. It is unclear if the modulations of J actually lead to level crossings (i.e. is J_min comparable to a in the cases where large enhancement is seen). Also it is not clear from the discussion around Eq. (10) why particular frequencies ν_d cause large enhancements for a particular J_0, i.e. why is there an apparent resonance effect. It is not clear to me that a Landau-Zener type mechanism would lead to this resonance-like effect.

Reviewer: 2

Comments to the Author

During the last decade, the radical pair model of the putative molecular avian compass has received growing attention in the scientific literature. The senior author of the present paper is particularly active in this area, with about 14 papers on this subject since 2016.

In the present manuscript, the focus was on the magnetic compass sensitivity enhancing effect of a harmonically driven distance variation of the active radical pair in model systems of varying complexity, mimicking the physical situation in the cryptochrome based magnetic compass. Such a motion modulates the sizes of exchange interaction, electronic magnetic dipole-dipole interaction, and electron transfer rate constant of radical pair recombination. The pertinent master equation for a harmonically driven radical pair distance was solved by applying the Floquet theory formalism. It was shown that, essentially due to the modulation of exchange interaction, the sensitivity of the magnetic compass, which is reduced by static exchange interaction, is again recovered, or even enhanced in certain frequency regions of the driving motion.

In principle, the positive effect of time-varying exchange interaction on the compass sensitivity is not new, as documented by pertinent references in the paper, the specifically novel aspect being associated with the driven harmonic variation. But it is just that aspect, that needs to be justified with respect to the realisitc natural system. Several arguments for the heuristic value of the present study and perspectives of its further development are presented in the discussion. These future scenarios (damped and over-damped Brownian motion) come close to the MD simulation of Ref. (32) and its consequences for enhancing the performance of a cryptochrome-based magnetic compass sensor. There, the motional effects have been condensed into a simple rephasing term in the master equation. The implications of that work should be given specific attention in the discussion of the present paper.

Minor points:

- The concrete figure of the radical pair separation r0 (following from the coordinates given in the ESI) should be mentioned in the main text.

- Also given, e.g. in pertinent Figure legends, should be the value of the EED, preferably expressed as a D value.

- On page 7, the paragraph beginning with “A similar scenario arises for A||…

Is hard to understand. The way it is written, it seems to encompass conflicting statements.

- Legend to Figure 5a: the value of the modulation frequency \nu&0 should be also stated here.

Author's Response to Peer Review Comments:

Response to Reviewers

We thank the reviewers for their comments and suggestions for additions, actioning on which has allowed us to further broaden the scope and thereby improve our manuscript. Specifically, we have clarified the discussion regarding the feasibility and relevance of our simple model in view of realistic biological environments. Our following point-by-point reply addresses all points raised in detail.

Reviewer 1

Recommendation: This paper is publishable subject to minor revisions noted. Further review is not needed.

Comments:

In this paper the authors present a novel and interesting idea that driven oscillations of singlet radical pair separation can enhance anisotropic magnetic field responses in the well-studied FAD-Trp radical pair which has been suggested to form the basis of avian magnetoreception. Previous studies on realistic models of this system find very low anisotropic MFE responses of the FAD-Trp pair, so new suggestions for mechanisms by which larger anisotropies can be found are clearly needed to understand the long-standing mystery of magnetoreception.

The results, which show that deterministic driving forces perturbing the inter-radical distance, even when damped, can lead to significant enhancement of anisotropic MFEs are particularly interesting, and the study is thorough.

A concern of mine is that the model of deterministic r(t) seems very physically unrealistic for a biological system. I would suggest the authors move some discussion of physical motivation for using deterministic models to where the model is introduced and include more discussion of why this driven model was chosen over arguably more realistic stochastic models for example underdamped Brownian motion (presumably computational expense of Floquet theory vs the Stochastic Schrodinger equation?).

Response A: We appreciate the reviewer noting the need for including more nuanced discussions to justify our model. It is obvious, but by design, that our model entails simplicity that is not expected to be identically found in real biological systems. The study design here has been chosen with the following objectives in mind:

a) At this stage, we aim to identify strong underlying principles rather than model biological realization. In particular, we seek to identify unifying building blocks, simple notions, from which emergent properties, such as enhanced magnetic field sensitivity, can later result in complex systems. This mandates a reductionist approach, which in our case has led to the idea of a spin system coupled to a single, classical, harmonic mode. This model fills

in a gap in the study of systems subject to Brownian dynamics as a limiting case; the other extreme, overdamped motion, has been studied in [Kattnig 2016].

b) As the reviewer points out, our choice leads to models that are computationally tractable, via Floquet theory. This enables a systematic exploration of the relevant parameter space even for spin systems encompassing the strong coupled nuclei in both the flavin and the tryptophan radicals (Fig. 4 and 5). The simulation of the arguably more realistic underdamped Brownian motion via the stochastic Schrödinger equation (cf. Response B below), requires markedly more computational resources (for frequency of the order 10 MHz, by a factor of at least 100,000), as it requires sampling of the stochastic motional trajectory (at least 1000 independent trajectories) and simulations extending to ~10 radical pair lifetimes (instead of two periods of the harmonic motion).

c) We seek to inspire applications in quantum technology, where harmonic driving could be engineered, whilst being inspired by the notion of “live” biology, i.e. non-equilibrium systems, for which structured driving is actively maintained, rather than mere equilibrium protein dynamics. Such dynamics have for example been suggested to underpin protn pumps [Friedman2021]. In this context we remark that our approach can be viewed as a first step towards dissipative spin dynamics in a structured environment (i.e. with structured spectral density) in the sense of a (classical) reaction-coordinate mapping approach.

Clearly, our propensity to reduce to simplicity the nature of the driving, i.e. environment, must not lead to misleading interpretations of possibilities. We have therefore studied damped oscillations and now, following the suggestion by the reviewer, an example of underdamped Brownian motion, both of which support the concept of structured driving insofar as they surpass the prediction of the harmonic model for realistic parameters.

The overall suggestion of the reviewer is to include discussion of the physical motivation where the model is introduced. We have followed this recommendation and now included a brief discussion of the choice of model, explaining that:

“Following the idea of a reductionist approach, our model has been idealized by assuming periodic harmonic driving. Whilst real proteins may not respond, e.g. to charge separation, in this exact manner; our motivation is to identify fundamental enhancing properties of driven radical motion, that could potentially be harnessed in technology or mediated by dynamics in (artificial and natural) spin systems by actively maintaining a structured reaction coordinate. The simplicity of the model also enables efficient computer simulations via Floquet theory, which allows a systematic evaluation. More realistic models involving damped motion and underdamped Brownian motion are discussed below.”

Ideally I would like to see at least one example (i.e. one large value of J_0 e.g. ~10MHz, perhaps with the one nuclear-spin model of FAD-Trp) of using underdamped stochastic Brownian motion for r(t), instead of the undamped harmonic model used in this paper, using the stochastic Schrodinger equation.

Response B: We are pleased to accept the reviewer's suggestion and have added a simulation using underdamped stochastic Brownian motion to the manuscript. The figure below illustrates the spread of the singlet recombination yield for a Brownian oscillator of frequency 4.9 MHz and distance fluctuations of approximately 2 Å (details provided in updates Supporting Information). It is evident that for a velocity damping constant, η, of up to ~10 μs^{-1}, the compass sensitivity is enhanced over the static limit. More surprisingly, weak damping appears to enhance the sensitivity over the undamped harmonic limit (realized for $\eta \rightarrow 0$). Overall, this demonstrates that the principle of enhancements from radical motion discussed in the manuscript translates to the (potentially more realistic for biological systems) stochastic Brownian motion treatment for a radical pair with inter-radical interactions.

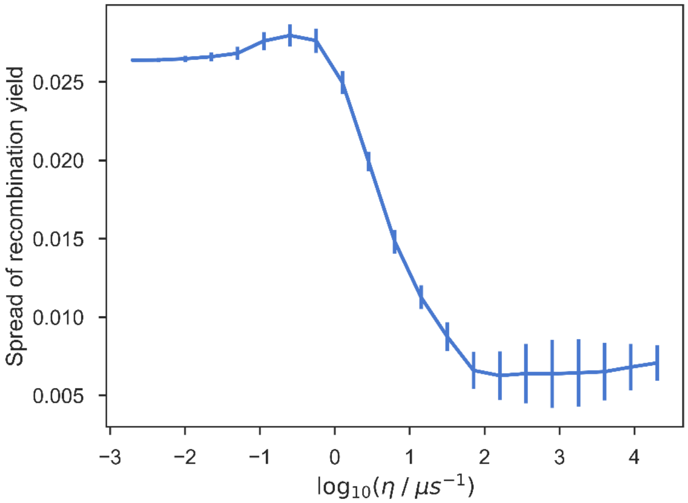

In view of these affirmative results, we have added to the discussion the following:

"In the SI we have additionally considered a more realistic treatment of protein mediated radical motion with a Brownian dynamics model implemented via the stochastic Schrödinger equation [Fay 2021] (see Fig. S13). This model calculation demonstrates that for weak velocity damping, the compass sensitivity can be enhanced over the static limit and even the idealized undamped harmonic model, suggesting that more realistic models could be better amplifiers. However, a comprehensive investigation of this, which is currently underway, is beyond the scope of the present study."

The authors should also clarify in their introduction what precisely they mean by "driven" in the context of their work - particularly if stochastically driven underdamped Brownian oscillator models produce similar enhancements to the undamped/deterministic cases, and clarify if/how this effect is different to previously described relaxation effects.

Response C: We use "driving" to indicate the presence of a strong determinist harmonic component in the distance modulation. Such motion could emerge intrinsically (e.g. as a result of relaxation oscillations in bistable systems or during the course of relaxation of a non-equilibrium system) or be actively maintained. A common feature is the non-Markovian character of the

resulting spin dynamics resulting from the motion. While underdamped Brownian oscillator models can produce enhancements (see above), we have here observed such enhancements only for weakly damped systems that fulfill the concept of “driving”, as introduced above. Note that Markovian dynamics (as emerging in the overdamped Brownian regime) can in fact too give rise to enhanced magnetic field effects for selected inter-radical interactions including involving significant exchange coupling (as previously described by in ref. [Kattnig 2016]), however these do not fulfill our definition and rely on alternative mechanisms (lifetime broadening of adjacent resonances).

We have now provided a definition of “driving” in line with the above statements. Our additions are summarized together with our reply to the subsequent, related point.

The concept of a "live" magnetoreceptor is also very loose and not particularly helpful - I suggest removing discussion of this as "live" model. If underdamped thermal Brownian motion can also produce enhanced anisotropy then I would argue that describing the enhancement effect as "live quantum sensing" is misleading.

Response D: While “driven” and “live” are not necessarily congruent, the structured driving is discussed here is arguably more likely to be realized for an actively maintained non-equilibrium system, thus fulfilling criteria of “live” systems. While underdamped Brownian motion can certainly ensue in various “dead” contexts, underdamped oscillations in a biological system are rare. In fact, “live” is inspired here by preceding works of Cai, Popescu, and Briegel, who coined the term in the context of ``live'' entanglement, for similarly driven phenomena that are persistent, dynamically controllable and exists only while metabolic processes take place, i.e. while the system is maintained far from thermal equilibrium in open driven systems [Cai 2010, Mohseni 2014, Zwanzig 2001]. Here we have retained this definition for quantum processes in the context of sensing in magnetoreception.

A paragraph in the introduction has been updated to reflect this argument and provide clarification of “driven” in relation to stochastic fluctuations, as requested above, and the definition of “live” quantum processes, which we wish to retain in order to link to prior studies carried out by Briegel et al. The combined paragraph, including a few original statements for context, now reads:

“It has been established that stochastic fluctuations of inter-radical distances due to (equilibrated) molecular dynamics can demonstrate improved magnetic sensitivity through spin relaxation in the Markovian limit [Kattnig 2016]. However, the question remains if enhancements could arise from driving, i.e. structured molecular dynamics that imprint a time-dependence on the inter-radical separation. Here, we use “driving” to indicate the presence of a strong deterministic component in the distance modulation. Such motion could emerge intrinsically (e.g. as a result of relaxation oscillations in bistable systems or during the course of relaxation of a non-equilibrium system) or be actively maintained or even realized via potentially artificial means. A common feature is the non-Markovian character of the resulting spin dynamics resulting from the motion. In the context of entanglement, Cai, Popescu, and Briegel have previously associated similar driving with a class of ``live'' phenomena that are persistent, dynamically controllable, and exists only while metabolic

processes take place, i.e. while the system is maintained far from thermal equilibrium in open driven systems [Cai 2010, Mohseni 2014, Zwanzig 2001]. Here we retain this definition for "live" magnetoreception. To investigate the possibility of enhancements arising from driven radical motion, under this definition, we extend on the established RPM to incorporate inter-radical distance modulation, approximated as harmonic motion that modulates the exchange/EED interactions and the recombination rate, to find that the magnetic field sensitivity can be vastly amplified."

Also it is well-established that when the FAD-Trp radical pair system is driven by radio-frequency magnetic field noise then anisotropy in the magnetic field response is suppressed, so I think it should be clarified in the title to reflect this. The title currently suggests to me that arbitrary driving can enhance sensitivity which is not the case.

Response E: We appreciate Reviewer 1 noting the importance of the effects of "radio-frequency magnetic field noise" being suppressive to sensitivity. We agree that the enhancements do not simply arise out of the effects of arbitrary driving, and have attempted to clarify this by modifying the title to "Driven radical motion enhances cryptochrome magnetoreception: Towards live quantum sensing" to avoid misconception with arbitrary driving. Similar changes have been made throughout the manuscript where misconception might arise.

I also believe the discussion around equation (10) explaining the origin of the enhanced anisotropic needs further expansion. It is unclear if the modulations of J actually lead to level crossings (i.e. is J_min comparable to a in the cases where large enhancement is seen). Also it is not clear from the discussion around Eq. (10) why particular frequencies v_d cause large enhancements for a particular J_0, i.e. why is there an apparent resonance effect. It is not clear to me that a Landau-Zener type mechanism would lead to this resonance-like effect.

Response F: We thank the reviewer for raising this point, which has motivated us to elaborate and expand our justification for the origin of the anisotropy enhancements. The pertinent points are that the modulation of J indeed takes the system through level-crossings. This does not necessitate that J_{min} is comparable to a, but that $J(t)$ comprises such values. The observed phenomena is then a prototypical harmonically driven Landau-Zener scenario, which has also been referred to as driven Landau-Zener-Stuckelberg-Majorana transition. The resonance like features can be understood as a consequence of the adiabatic evolution of the system in between diabatic transitions when transitioning the crossing. The adiabatically accumulated phases between transitions can constructively or destructively interfere in the diabatic evolution, thereby leading to the observed resonance-like features as J_0 and ν_d are varied. A detailed discussion and quantitative description of this is e.g. provided in [Shevchenko 2010], which is now cited.

We have made the following changes in the main manuscript:

First, we have provided a quantitative assessment of the modulations of J that demonstrate intermittent values are comparable to a for parameters considered in the study: "Driving this system through a modulation of the inter-radical distance introduces a time dependent $J(t)$,

decreasing it from $J(t) = J_0$ at $t = n/\nu_d$ to $J(t) = J_0 \exp(-\beta\, \Delta_d)$ at $t = (2n+1)/2\nu_d$ for $n \in \mathbb{Z}^+$, thereby driving the system through avoided crossings for $a \sim J(t)$."

Second, we have adopted the nomenclature "Landau-Zener-Stuckelberg-Majorana (LZSM)" to refer to the transitions as this links the phenomenon to literature (e.g. Shevchenko et al.) which discusses these transitions in the more general setting of periodic driving.

Third, we discuss the origin of the observed resonances as a consequence of the accumulated adiabatic phases between transitions: "In turn this induces LZSM transitions from $|S\rangle$ to $|T_0\rangle$, which can be described as adiabatic evolutions interrupted by diabatic transitions as the system transitions through the anti-crossings [Shevchenko 2010]. The adiabatic phases accumulated between transitions can constructively or destructively interfere in the subsequent diabatic transition event, leading to the observed resonance effects as J_0 and ν_d are varied."

We also provide reference to a result in the SI demonstrating that the resonance effect persists for a simpler models: "In the SI we confirm that general enhancement features persist for a further simplified two-level system which, under a small amplitude expansion of the driving, is akin to the model considered by Shevchenko et al. [Shevchenko 2010] (see Fig. S8)", and as a conclusion "The basis of this principle extends to systems with more hyperfine couplings and $A_\perp \neq 0$."

Reviewer 2

Recommendation: This paper is publishable subject to minor revisions noted. Further review is not needed.

Comments:

During the last decade, the radical pair model of the putative molecular avian compass has received growing attention in the scientific literature. The senior author of the present paper is particularly active in this area, with about 14 papers on this subject since 2016.

In the present manuscript, the focus was on the magnetic compass sensitivity enhancing effect of a harmonically driven distance variation of the active radical pair in model systems of varying complexity, mimicking the physical situation in the cryptochrome based magnetic compass. Such a motion modulates the sizes of exchange interaction, electronic magnetic dipole-dipole interaction, and electron transfer rate constant of radical pair recombination. The pertinent master equation for a harmonically driven radical pair distance was solved by applying the Floquet theory formalism. It was shown that, essentially due to the modulation of exchange interaction, the sensitivity of the magnetic compass, which is reduced by static exchange interaction, is again recovered, or even enhanced in certain frequency regions of the driving motion.

In principle, the positive effect of time-varying exchange interaction on the compass sensitivity is not new, as documented by pertinent references in the paper, the specifically novel aspect being associated with the driven harmonic variation. But it is just that aspect that needs to be justified with respect to the realistic natural system.

Response G: It is true that a previous work [Kattnig 2016] has addressed the effects of Markovian fluctuations of inter-radical interactions and found them enhancing on the compass sensitivity under certain conditions. However, this enhancing effect did not extend to the important $J = 0$ scenario and the degree of enhancement were comparable to what could be realized by a suitable chosen static J, as the effect relies predominantly on the broadening of resonances of adjacent Js. This posits that the effect will be reduced in more complex systems than those studied in [Kattnig 2016]. The effect considered here can deliver true enhancements, applies to $J = 0$ (even for under-damped Brownian dynamics; see Response B) and extends to large J values and large spin systems of comparable hyperfine structure.

The question about the relevance of the chosen model of driven harmonic variation for natural systems has also been raised by Reviewer 1. We shall reiterate the most pertinent points here and refer to Response A and Response B for a more detailed discussion and the changes made to the manuscript. In short, the model was chosen for reductionist simplicity, out of consideration for computational efficiency (solutions amenable to the Floquet approach) and with the aim to encompass "live" processes, i.e. non-equilibrium oscillation relaxation and actively maintained structured motion. With respect to the last point, we follow up on ideas of Cai, Popescu, and Briegel, who have previously suggested ``live'' entanglement as a consequence of similar open system dynamics [Cai 2010, Mohseni 2014, Zwanzig 2001]. Clearly, realistic natural systems will not adhere to the single-harmonic-mode picture studied, but we expect that these motional characteristics are contained in the motional response of cryptochrome to perturbations, such as the charge separation following light activation, overlaid on a background of Markovian, overdamped dynamics. Our first explorations using damped harmonic oscillations and the, now added, Brownian dynamics, suggest that the effects are sustained—even enhanced—under more realistic scenarios.

Several arguments for the heuristic value of the present study and perspectives of its further development are presented in the discussion. These future scenarios (damped and over-damped Brownian motion) come close to the MD simulation of Ref. (32) and its consequences for enhancing the performance of a cryptochrome-based magnetic compass sensor. There, the motional effects have been condensed into a simple rephasing term in the master equation. The implications of that work should be given specific attention in the discussion of the present paper.

Response H: The model studied in [Kattnig 2016] and the model elaborated here correspond to the opposite limiting cases of Brownian motion in the limit of strong and weak damping, respectively. While the effect of overdamped (and thus Markovian) dynamics can be well accounted for by a simple dephasing term in the master equation, this is not possible for the driven dynamics studied here. In any case, as both limiting cases can be enhancing in principle, this

suggest that indeed the envisaged study of the intermediate damping regime will be auspicious. For $J = 0$, we have now already undertaken such a study for a simple system (please see Response B), the conclusion being that for this particular coupling only driven systems are strongly enhancing (as expected; see Response G). The study of molecular dynamics will be useful in future to inform the characteristic of radical pair motion. However, it must be realized that MD studies so far have only considered equilibrium scenarios and have been too short to reveal oscillatory components in the lower MHz frequency range. This is the subject of an intended study of ours.

We have further detailed the relation to Ref. 32 and stochastic fluctuations of the equilibrated MD that results in a noise assisted spin relaxation mechanism in our introduction (see Response C) and in our discussion where we have added:

“It has been established that stochastic fluctuations of inter-radical distances due to the equilibrated molecular dynamics can demonstrate improved magnetic sensitivity through spin relaxation in the Markovian limit [Kattnig 2016]. However, the question remains if enhancements could arise from driving, i.e. structured molecular dynamics that imprint a time-dependence on the inter-radical separation.”,

and the following at suitable locations:

“By maintaining relevant quantum systems in a ``live'' far from equilibrium state, driving may constitute a crucial addition to noise assisted processes.”

“In fact, the model studied in [Kattnig 2016] and the model presented here correspond to the limiting cases of Brownian motion in the limit of strong and weak damping, respectively."

"Oscillatory motion resulting from the photo-activation of cryptochrome could be identified by molecular dynamics simulations, which so far have only been used to study equilibrium configurations and for timescales insufficient to assess the desired frequency spectrum [Schuhmann 2021, Kattnig 2016].”

Minor points:

- The concrete figure of the radical pair separation r0 (following from the coordinates given in the ESI) should be mentioned in the main text.
- Also given, e.g. in pertinent Figure legends, should be the value of the EED, preferably expressed as a D value.
- On page 7, the paragraph beginning with “A similar scenario arises for A||…
Is hard to understand. The way it is written, it seems to encompass conflicting statements.
- Legend to Figure 5a: the value of the modulation frequency \nu&0 should be also stated here.

Response H: We have added in the radical pair separation r_0 at initial time/for the static radical pair model at the point which it is discussed.

The D value for EED interaction has been added to all figures where appropriate, as has the modulation frequency to Fig. 5a.

The explanation on page 7 has been restructured in tandem with Response E to provide a clearer assessment of the origin of enhancements in the range of large to moderate J_0-values. Specifically, we write: "…a similar expression applies for the parallel orientation (see SI). The scenario encoded in these representation matrices resembles a Landau-Zener-Stückelberg-Majorana (LZSM) transition"

Non-scientific changes:

We have addressed all of the following editorial changes and further detail is provided where necessary:

1) Title: In both the main manuscript file and the Supporting Information, set the title in title case, with the first letter of each principal word capitalized.

2) Figures: Please consider displaying Figure 1 in color.

Figure 1. has been improved to include a color coordinated reaction scheme in correspondence with a protein structure that shows the relevant radical pair and inter-radical distance.

3) TOC Graphic: Please resize the TOC graphic per journal guidelines (2 in x 2 in) and move to the correct position (on the same page as the abstract).

4) Supporting Information: Please add full header at top of page of the Supporting Information file, which includes: Title, Full Author List, and Author affiliations (exactly as they appear in the manuscript).

5) References: In both the main file and the supporting information, fix the style of all references to use JPCL formatting (check all references carefully). ***JPC Letters reference formatting requires that journal references should contain: () around numbers, author names, article title (titles entirely in title case or entirely in lower case), abbreviated journal title (italicized), year (bolded), volume (italicized), and pages (first-last). Book references should contain author names, book title (in the same pattern), publisher, city, and year. Websites must include date of access.

6) Supporting Information: Please number SI pages in the following format: "S1, S2…"

Concluding Remarks

Overall, we would like to stress that our results showcase enhancements in sensitivity that arise from a very general form of inter-radical distance modulation, the origin of which can arguably be biological, but could certainly also be engineered and/or artificially enhanced.
